# Inflammation and Psoriasis: A Comprehensive Review

**DOI:** 10.3390/ijms242216095

**Published:** 2023-11-08

**Authors:** Alessandra-Mădălina Man, Meda Sandra Orăsan, Oana-Alina Hoteiuc, Maria-Cristina Olănescu-Vaida-Voevod, Teodora Mocan

**Affiliations:** 1 Physiology Department, Faculty of Medicine, “Iuliu Hatieganu” University of Medicine and Pharmacy, 400126 Cluj-Napoca, Romania; madalina.man@outlook.com (A.-M.M.); hoteiuc.oana@gmail.com (O.-A.H.); cristina.vaida2008@gmail.com (M.-C.O.-V.-V.); 2 Physiopathology Department, Faculty of Medicine, “Iuliu Hatieganu” University of Medicine and Pharmacy, 400126 Cluj-Napoca, Romania; orasan.meda@umfcluj.ro; 3 Nanomedicine Department, Regional Institute of Gastroenterology and Hepatology, 400158 Cluj-Napoca, Romania

**Keywords:** psoriasis, biologics, IL-17, IL-23, TNF-α, biomarkers, nanotechnology

## Abstract

Psoriasis is an immune-mediated disease with a strong genetic component that brings many challenges to sick individuals, such as chronic illness, and which has multiple associated comorbidities like cardiovascular disease, metabolic syndrome, inflammatory bowel disease, and psychological disorders. Understanding the interplay between the innate and adaptative immune system has led to the discovery of specific cytokine circuits (Tumor Necrosis Factor-alpha (TNF-α), IL-23, IL-17), which has allowed scientists to discover new biomarkers that can be used as predictors of treatment response and pave the way for personalized treatments. In this review, we describe the footprint psoriasis leaves on the skin and beyond, key pathophysiological mechanisms, current available therapeutic options, and drawbacks faced by existing therapies, and we anticipate potential future perspectives that may improve the quality of life of affected individuals.

## 1. Introduction

Psoriasis is a chronic, inflammatory, autoimmune disease that affects the skin and predominantly occurs in individuals with a strong genetic predisposition [1].

In 2014, the WHO recognized psoriasis as a “chronic, non-communicable, painful, disfiguring and disabling disease for which there is no cure and with a great negative impact on patients’ quality of life” due to the huge emotional, social, and economic burden of the disease [2].

### 1.1. Epidemiology Data

To date, the prevalence of psoriasis is only known in approximately one-fifth of all countries, ranging from 0.14% in East Asia to 1.99% in Australasia. Psoriasis is not equally distributed in all geographical areas, with its prevalence being closely linked to ethnicity. It is found to be most common among Caucasian people living in Central and Western Europe (1.83% and 1.92%, respectively) and North America (1.50%) [3,4]. Less is known about the incidence of the disease, with current reports coming mainly from high-income countries in Europe (mostly the UK) and North America. Psoriasis can start at any age, but there are usually two peaks for the onset of the disease. Men typically have an onset of disease between 30–39 years or 60–69 years, and women typically have an onset between 18–29 years or 50–59 years. There is also an increasing prevalence in the pediatric population, from 0.13% in 0–2-year-olds and 0.67% in 14–18-year-olds, suggesting that psoriasis is not frequent in children. There is also a trend in the decreasing incidence rate of psoriasis that could be explained by people with the disease living longer due to early diagnosis and treatment. Patients still, however, have a shorter life span than the healthy population due to disease-associated comorbidities [5]. The prevalence of psoriasis is equal in both sexes, but men have a more severe form of the disease compared to women, as reported by Hagg et al. This can explain the fact that men receive systemic biological therapy for psoriasis more frequently than women [6].

### 1.2. Genetic Background, Epigenetic Modifications, and CRISPR/Cas-9 Genome Editing

Psoriasis is one of the cutaneous diseases that has been greatly investigated in Genome-Wide Association Studies (GWAS) in the last two decades, with the initial whole genome sequencing and analysis conducted in 2005 [7].

Heritability is an important factor in the development of the condition, since the incidence of psoriasis is obviously higher in first-degree relatives of patients (around one-half of their genetic information is shared with parents, siblings, and offspring) and among monozygotic twins compared to dizygotic twins, the latter being three to four times lower in terms of risk (65–72% versus 15–30%) [8].

Sixty-three susceptibility loci have been discovered so far in European individuals, which explains 28% of the heritability of the disease [9].

The genetic background of psoriasis covers nine genomic susceptibility regions (*PSORS 1–9*) and, as previously reported, HLA-C*06:02 (*PSORS 1*) is the most likely allele linked to susceptibility, disease severity, comorbidities, phenotypic features (early-onset, guttate form), and variability in treatment response. *PSORS1* lies in the Major Histocompatibility Complex (MHC) class I on chromosome 6p21 and mainly comprises genes with an important role in antigen presentation. Moreover, in individuals carrying the HLA-C*06:02 allele, if Endoplasmic Reticulum Aminopeptidase I (ERAP I) is additionally present (which is an important milestone in MHC class I peptide processing, coding an aminopeptidase), it substantially increases the risk of individuals developing the disease [8,10].

Data regarding the correlation between *HLA-Cw6* (HLA-C*06:02) status and response to biological therapy are spare and contradicting. Dand et al. conducted a study to test if the HLA-C*06:02 allele is a predictive biomarker of biological treatment response for two drug agents commonly prescribed to patients with moderate to severe psoriasis resistant to conventional therapeutic protocols: Adalimumab, an anti-TNF-α drug, and Ustekinumab, a monoclonal antibody binding the p40 subunit, shared by IL-12 and IL-23. This study stated that HLA-Cw6-negative patients had a better response to Adalimumab than Ustekinumab and that HLA-Cw6-negative patients also positive for arthritis were associated with an even better response to treatment [11]. Similar findings were reported by Burlando et al., who evaluated the effectiveness (namely a reduction in Psoriasis Area Severity Index (PASI) score of 90%) of three different classes of biological agents: TNF-α, IL-17, and IL12/23 inhibitors at weeks 16 and 48 of use. At week 16, all agents showed efficacy with no statistically significant difference, but at week 48, the HLA-Cw6-positive patients treated with anti-IL-17 or anti-IL12/23 drugs responded better. Thus, Cw6 seems to be a predictor of treatment response at week 48, with anti-TNF-α drugs being more effective on Cw6-negative individuals undergoing biological therapy [12]. Contradictory to this, Constanzo et al. reported a high PASI 90 score in patients treated with Secukinumab (IgG1ᴋ monoclonal antibody that binds interleukin 17A), regardless of the HLA-Cw6 status. This shows that although HLA-Cw6-positive and negative patients have different clinical features of the disease, they achieve the same clinical response, and thus testing for HLA status before initiating Secukinumab is not a useful tool [13].

HLA-Cw6-positive status is also linked to extensive skin involvement and is associated with large plaques on the arms, legs, and trunk [14]. Additionally, the autoantigens cathelicidin (LL37) and ADAMTSL5, abundantly expressed in injured skin, bind to HLA-Cw6 with high affinity. Thus, psoriasis patients harboring the *HLA-Cw6* allele are at higher risk of developing lesions following minor trauma (Koebner phenomenon) [15].

Other studies have reported that HLA-Cw6-positive patients had less frequent nail disease and scalp involvement [16,17,18]. This is very important since the inflammation of the nail bed, which shares a common tendinous insertion with the distal interphalangeal joint commonly affected by arthritis, might be predictive of a higher incidence of psoriatic arthritis. Furthermore, scalp lesions may be associated with arthritis, given the abundant microbial flora at these lesion sites, serving as a doorway for microorganisms to trigger an abnormal immune response related to the pathogenesis of psoriasis.

As psoriasis is a heterogeneous condition, several clinical subtypes have been of interest to researchers, specifically guttate and pustular psoriasis. Guttate psoriasis is usually diagnosed in children and young adults diagnosed with *Streptococcus pyogenes* pharyngitis or tonsilitis, becoming symptomatic after a mean period of two weeks. Mallon et al. studied the association between the HLA-C locus and guttate psoriasis using high-resolution Polymerase Chain Reaction-Single Specific Primer (PCR-SSP) and found a direct link between HLA-Cw6-positive status and the pathogenesis of guttate psoriasis (all subjects with guttate psoriasis carried the allele, compared to 20% of the control group) [19].

The term pustular psoriasis is used to describe a subset of rare clinical phenotypes, ranging from acute, Generalized Pustular Psoriasis (GPP) to chronic, localized Palmoplantar Pustulosis (PPP) and Acrodermatitis Continua of Hallopeau (ACH), all of which are characterized by neutrophilic sterile pustules eruption. Pustular psoriasis usually accompanies psoriasis vulgaris, but the striking clinical, genetic, and histopathological differences suggest that it is more likely to be a distinct entity [20]. The IL-1 cytokine family comprises IL-36RN and plays an important role in innate immunity through a firm, strong, and non-specific response to pathogens. IL-36RN and its paralogs (namely IL-36A, IL-36B, IL-36G) are generously expressed in the skin, and the proteins encoded by these genes interact with the IL-12RL2 receptor to modulate Nuclear Factor- ᴋB (NF-ᴋB) signaling. IL36RN specifically blocks the activity of IL-36A and IL-36G by preventing the recruitment of the IL-1RL2 receptor complex. Onoufriadis et al. reported that the loss of function of IL-36RN is the key for developing GPP, but it is not responsible for the development of PPP [21]. GPP was also found to be related to mutations in the *CARD14* gene-encoding for a keratinocyte nuclear factor ᴋB adaptor protein and the *AP1S3* gene, which encodes a subunit of the adaptor protein 1 complex [22,23]. On the opposite end of the spectrum, PPP, which is more frequent in women with a history of cigarette smoking, is associated with *CARD14* missense variants but not with IL-36RN mutations [24].

Secondly, even though other variants do not have such a great genetic impact, they highlight the pivotal role of innate and adaptative immunity in disease pathogenesis and facilitate the development of new and targeted therapies, with the IL23/Th17 cytokine circuit being the primary target of choice. Crucial pathways and their candidate genes include those involved in NF-ᴋB signaling (*TNIP1*), the type 1 interferon pathway (*IFIH1*), skin barrier function (*LCE3*), and last but not least the IL-23/Th17 axis (IL23R, IL12B, TYK2) [25].

The dynamic regulation of the psoriasis genome beyond genetic variations is highlighted by epigenetic modifications. Epigenetic factors can regulate gene expression at the transcriptional (histone modification, DNA methylation) and posttranscriptional levels (microRNA and long non-coding RNAs) without affecting the DNA sequence [26].

The DNA methylation process is defined as a covalent binding of methyl groups to cytosine from CpG islands in the promoter region of genes. This bond restricts the access of DNA transcription factors, resulting in the silencing of gene function [26]. Considering the role of effector T-cells in psoriasis and Psoriatic Arthritis (PsA) pathophysiology, a research paper published by Valentina Natoli et al. identified some DNA methylation patterns in CD4+ T cells that could contribute to the discrimination process between psoriasis patients and controls. She found the following patterns: CpG site cg01877366 located in Trafficking Protein Particle Complex Subunit 9 (*TRAPPC9*), cg1120622 in Reversionless 3-like (*REV3L*), and cg18925478 in Phosphatase and Actin Regulator 2 (*PHACTR2*). *TRAPPC9* was reported to be a predictive marker for failure in patients treated with TNF-α inhibitors and *REV3L* was found to be a candidate target gene for psoriasis and PsA treatment/therapy. *PHACTR2* was also linked as a marker for systemic lupus erythematosus. Another major finding of this study states that biological agents (IL-17 and TNF-α inhibitors) can modulate the DNA methylation process of IL-17 and TNF corresponding genes, and that there is an inverse correlation between DNA methylation and PASI score [27].

Histone modifications have also been described to be implicated in psoriasis pathogenesis. H3K9me2 modifies IL-23 expression in keratinocytes. The inhibition of Enhancer of Zeste Homolog 2 (EZH2), a H3K27 methylase, causes H3K27me3 downregulation and subsequently suppresses the hyperproliferation of the epidermis in murine models. The inhibition of Histone Deacetylase-1 (HDAC), which is upregulated in psoriatic skin, regulates T reg function by preventing the production of IL-17 [28]. Moreover, following biological treatment, significant differences in methylated H3K27 and H3K4 were found between responders (PASI 75) and non-responders [29].

Lastly, several studies have been conducted to elucidate the role of miRNAs in psoriasis. MiR-223 and miR-143 have the potential to discriminate between psoriasis patients and healthy controls and are positively correlated with PASI scores, which means that they can be used as diagnostic and disease severity assessment biomarkers. With respect to therapeutic monitoring, miR-146a-5p and miR-125a plasma levels have been reported to correlate with the efficacy of Adalimumab and Etanercept treatments, respectively. MiRs were also evaluated as therapeutic agents in in vivo and mouse models studies, with promising results. They observed that the inhibition of miR-21 alleviated disease severity and reduced epidermal hyperplasia, and that the topical administration of nanocarrier miRNA-210 antisense blocked the development of psoriasis-like dermatitis [30].

Gene therapy has become a promising approach in psoriasis, with CRISPR-Cas9 becoming one of the most remarkable and widely used methods. Arakawa et al. generated ERAP-1 knockout melanoma cell lines (WM793 or WM278) by CRISPR/Cas-9 genome editing and revealed that the cell surface expression of HLA-C*06:02 decreased significantly, leading to a lower density of self-peptide (ADAMTSL5)/HLA-C*06:02 complexes on the cell surface of the autoimmune target cells, thus reducing CD8+ T cell autoreactivity [31]. Another study conducted by Swindell et al. profiled the transcriptome of epidermal keratinocytes treated with IL-1B, IL-36A, IL-36B, and IL-36G and identified that shared IL-1B and IL-36 DEGs overlapped significantly with genes altered in psoriasis vulgaris and GPP skin lesions. They also showed that the MyD88 adaptor protein mediates shared IL-1B and IL-36 responses, and its inactivation using CRISPR/Cas9 eliminated the responses to IL-1B and IL-36 stimulation [32].

### 1.3. Triggers

Psoriasis occurrence is dependent on the interaction between genes and environmental factors in that the disease does not manifest unless some intrinsic or extrinsic triggers are present. Table 1 shows the link between the main triggers driving pathogenesis and the pathophysiological mechanisms that lead to the onset or flare of psoriasis.

**Table 1:** Link between triggers incriminated in psoriasis occurrence and pathophysiological mechanisms.

Trigger	Pathophysiological Mechanism	References
Mechanical stress	Nerve growth factor (NGF) associated with the Koebner phenomenon; upregulation of NGF in keratinocytes stimulates their proliferation	[33]
	Tissue resident memory T cells (T RM), site-specific memory T cells in the skin	[34]
	Skin injury induces IFN-β secretion from keratinocytes, promoting Dendritic Cell (DC) maturation and T cell proliferation	[35]
Psychological stress	Corticotropin-releasing hormone (CRH) that causes the degranulation of mastocytes and the release of TNF-α and IL-6	[36]
	High levels of corticosteroids (CS) stimulate 5-HT synthesis, increase 5-HT2AR (proinflammatory role, 5-HT-mediated recruitment of CD4+ lymphocytes), decrease 5-HT1AR (inhibitor of inflammation)	[37]
Infection	Streptococcal superantigens-pyrogenic exotoxin and M protein stimulate T cells to produce IFN-γ	[38]
	Molecular mimetism between M6 streptococcal protein and type I keratin	
	*Staphylococcus aureus* Enterotoxin-A (SEA) induces the production of IFN-γ in MHC class II positive T cell lines	[39]
	*TAT* gene promotes HIV replication and activates genes responsible for keratinocyte proliferation (IL-6, IL-2, TNF, TGFβ1, and Superoxide Dismutase (SOD))	[38]
	HIV causes an imbalance in the CD4+/CD8+ ratio, leading to a proinflammatory cascade characteristic of psoriasis. CD8+ T cells and memory CD8+ move up in the dermis of lesioned skin, resulting in exacerbation of psoriasis	[40,41]
	Gp120 and nef HIV proteins act as superantigens	[42]
	HPV infection triggers an inflammatory state that causes upregulation of NGF	[43]
Antibiotics	Tetracyclines photosensitize the skin, resulting in Koebnerization	[44]
	Amoxicillin: *Streptococcus pneumoniae* exposed to β-lactam antibiotics stimulates macrophage release of TNF-α and IL-1β	
Gut microbiome	Depletion of Short-Chain Fatty Acids (SCFA) antagonizes Treg differentiation and their suppressive activity and causes increased production of IL-23 via DCs	[45]
	“Leaky gut syndrome”: higher levels of E. coli DNA in psoriasis patients suffering from exacerbation causes activation of Th17 lymphocytes	[46]

### 1.4. Clinical Presentation

Psoriasis has five different clinical phenotypes, but the most frequent and most easily recognized is plaque psoriasis, also referred to as psoriasis vulgaris. Other less common forms of psoriasis include guttate, erythrodermic, pustular, and inverse/flexural psoriasis.

The classic morphology of chronic plaque psoriasis is that of sharply demarcated erythematous plaques covered by silvery scales. Removing the scales may result in small bleeding points, a fact known as the Auspitz sign. The plaques occur symmetrically in predilection sites like extensor surfaces of forearms and shins, the sacral region, retro-auricular area, and the scalp [47].

The scalp is the most common site of involvement at onset and throughout the disease (79% of patients) [48] and may cause increased shedding of hairs in the telogen phase, extensive hair loss, and even scarring alopecia in severe chronic hyperkeratotic subtypes [49].

More than 50% of patients with psoriasis have concurrent nail psoriasis, which may be a sign of more severe disease and a precursor of PsA, with the prevalence of PsA at 70–80% among individuals with nail involvement [50]. According to Zaias et al., pitting is the most common sign of nail psoriasis, followed by nail bed discoloration, onycholysis, subungual hyperkeratosis, modifications of the nail plate, and splinter hemorrhages. However, other studies have found that nail bed discoloration in the form of ‘‘oil drop’’ or ‘‘salmon-colored’’ spots is the rarest, reported in only 18% of the examined individuals [51].

## 2. Diagnosis

Diagnosis is usually based on clinical findings, but one major approach in understanding the mechanisms behind the disease and confirming the diagnosis is to perform a histologic and immunohistochemical examination of the affected skin [52].

### 2.1. Histopathology

Histopathologic findings in psoriasis vulgaris vary with the age and dynamics of the lesions. The earliest changes are nonspecific and consist of spare superficial perivascular T-lymphocyte infiltration with neutrophil exocytosis, followed by dermal edema and dilation of blood vessels within dermal papillae. The early stages of plaque formation further show epidermal hyperplasia, parakeratosis aggregates, and infiltration with lymphocytes, histiocytes, and neutrophils in the dermis. Mature plaques show important epidermal hyperplasia with characteristic features such as uniform elongation of the epidermal rete ridges and enlargement of their tips; dilated tortuous capillaries and fibrillary collagen (reciprocal elongation of dermal papillae); thinning of the suprapapillary plate; pallor of the superficial epidermis and minimal spongiosis, marked hyperkeratosis, parakeratosis, and subjacent hypogranulosis; and collections of neutrophils within the parakeratosis, specifically Munro’s microabscesses and less frequently within the spinous layer—spongiform pustules of Kogoj [53].

Histopathological and immunohistochemistry analysis may be a useful tool for decoding physiopathological mechanisms in psoriasis, such as the recruitment of circulating lymphocytes to inflammatory sites and their Lymphocyte Function-Associated Antigen 1 (LFA-1) dependent adhesion stimulated by endocan [54]. The cytotoxicity of CD8+ T cells and Natural Killer (NK) cells has also been found to be mediated by the perforin/granzyme pathway, explaining why perforin expression is upregulated in activated CD8+ T cells, which are abundantly expressed in the epidermis of psoriasis plaques [55]. Further, Simonetti et al. identified key factors in the link between inflammation and angiogenesis, specifically Vascular Endothelial Growth Factor (VEGF). They found that Hypoxia-Inducible Factor 1-alpha (HIF-1α) and Matrix Metallopeptidase 2 (MMP-2), which control the rate of *VEGF* gene transcription and play an important role in the remodeling of endothelial cell membranes, were both positively correlated with VEGF [56]. Moreover, different immunohistochemical studies found a correlation between psoriasis severity and Smad7 [55] and Plexin-B2 [57] expression. Smad 7 and Plexin-B2 are both activators of keratinocyte proliferation that act through distinct mechanisms. Firstly, the CD-100-Plexin-B2 complex activates NF-ᴋB and NLRP3 inflammasome in keratinocytes [58], while Smad7 inhibits the TGF-β1 signaling pathway, which normally directly inhibits keratinocyte growth [59] (inhibitor of the inhibitor). Lastly, immunohistochemistry reveals new targets for the development of therapeutic agents. It was found that the Fib3 antibody blocked the Fib3 function of promoting VEGF production, keratinocyte proliferation, and migration [60]. Studies evaluating candidate biomarkers in psoriasis through immunohistochemical analysis are listed in Table 2.

**Table 2:** Studies evaluating candidate biomarkers in psoriasis through immunohistochemical tissue analysis.

Author, Year	Study Design	Population	Comparison (Control)	Technique	Biomarker Levels/ Outcomes	*p*
[54]	Cross-sectional	Chronic plaque psoriasis patients *n* = 36	Normal individuals attending plastic surgery outpatient clinic *n* = 40 Uninvolved skin from 10 patients	Skin biopsy-3 mm punch, immunohistochemical staining of endocan	Upregulation of endocan in the involved psoriatic skin (63.9% in the epidermis and 36.1% in the dermis) and absence of its expression in uninvolved skin biopsies	
					Significant difference between psoriatic and normal skin in epidermal and dermal endocan expression	Epidermal endocan expression *p* = 0.003, dermal *p* < 0.001
					Strong epidermal expression of endocan in cases affecting the trunk compared to cases affecting extremities	*p* = 0.023
					Strong and moderate intensity of endocan expression by epidermis associated with marked acanthosis	*p* = 0.038
					Positive dermal endocan expression associated with marked parakeratosis and moderate angiogenesis	*p* = 0.018 parakeratosis *p* = 0.011 angiogenesis
[57]	Cross-sectional	Chronic plaque psoriasis *n* = 30	Normal individuals *n* = 20 Perilesional psoriatic skin *n* = 30	Immunohistochemical evaluation of Plexin-B2	Significant overexpression of Plexin-B2 in epidermal keratinocytes from controls to perilesional and lesional skin	*p* < 0.001
					Overexpression of Plexin-B2 in dermal inflammatory cells of lesional psoriatic skin compared to control	*p* < 0.001
					Positive correlation between Plexin-B2 H-score values in the lesional epidermis and PASI scores and in dermal inflammatory cells and PASI score	*p* < 0.001 *p* < 0.026
					High Plexin-B2 expression in lesional epidermis associated with parakeratosis	*p* < 0.016
					High Plexin-B2 expression in lesional dermal inflammatory cells associated with acanthosis and hyperkeratosis	*p* = 0.006 *p* < 0.001
[61]	Cross-sectional	Chronic plaque psoriasis *n* = 30	Normal individuals *n* = 20 Perilesional psoriatic skin *n* = 30	Immunohistochemical analysis of Smad7	Smad7 was progressively upregulated in proliferating keratinocytes from controls to perilesional and lesional skin	*p* < 0.01
					Dermal inflammatory cells showed up-regulation of Smad7 expression in lesional skin	*p* < 0.10
					Positive correlation of Smad7 expression with psoriasis severity	*p* < 0.012
[55]	Cross-sectional	Chronic plaque-psoriasis *n* = 11	Healthy *n* = 8 Perilesional *n* = 11	Immunohistochemistry -analysis of perforin, T, and NK-cells subsets	Significant increase in CD4+ and CD8+ cells in psoriatic skin compared with perilesional and healthy skin	*p* < 0.01 *p* < 0.001
					Expression of CD16+ NK cells lower in lesions compared with healthy skin	*p* < 0.05
					Perforin expression was significantly enhanced in the epidermis of psoriatic lesions compared to the dermis	*p* < 0.01
[56]	Cross-sectional	Chronic plaque psoriasis *n* = 15	Normal skin *n* = 5	Immunohistochemical study	In the epidermis of psoriatic skin, HIF-1α was diffusely expressed	
					Positive correlation between HIF-1α and VEGF in psoriatic skin	*p* = 0.010
					Total MMP-2 expression of healthy skin was significantly lower compared to psoriatic skin	*p* = 0.0001
					Positive correlation between VEGF and MMP-2 in psoriatic skin	*p* = 0.046
					Decreased expression of Tissue Inhibitor of Metalloproteinase-2 (TIMP-2) and TIMP-1 in psoriatic skin	*p* = 0.0001
[62]	Longitudinal	Chronic plaque psoriasis *n* = 27	Normal skin *n* = 20	Antimicrobial peptides (AMP) immunohistochemistry	The Human Beta Defensin-1 (hBD-1) level was higher in psoriasis patients than in healthy controls	*p* = 0.001
					No difference in hBD-1 and hBD-2 levels before and after phototherapy in patients’ group	
[60]	Longitudinal	Chronic plaque psoriasis *n* = 31 (tissue bank)	Normal skin *n* = 32	Immunohistochemistry Fib3, Western blot analysis, RT-PCR for Fib3mRNA expression	Fib3 overexpressed in the lesional skin; levels positively correlated with psoriasis progression	*p* < 0.05
					Fib3 upregulated VEGF expression in endothelial cells	*p* < 0.05
					Topical/ subcutaneous injection of Fib3 ab decreased PASI and VEGF expression in imiquimod-treated mice	*p* < 0.05 for both
[63]	Cross-sectional	Chronic plaque psoriasis *n* = 24	Healthy controls *n* = 12	Immunohistochemical serotonin expression	Serotonin expression was higher in psoriatic plaques compared to normal skin	*p* = 0.018
					H score of serotonin expression was higher in cases with absent Granular Cell Layer (GLC) and in cases with strong epidermal inflammation	*p* = 0.011 *p* = 0.035

### 2.2. Novel and Non-Invasive Techniques for Diagnosis and Therapeutic Monitoring

Advanced high-definition imaging techniques may be useful in the study of in vivo skin properties, in establishing a definitive diagnosis, and in the therapeutic monitoring of plaque psoriasis. Available imaging instruments vary in several parameters, such as resolution, depth of penetration, visual representation (horizontal, vertical, three-dimensional), and in the type of visualized skin structures. These include dermoscopy, videocapillaroscopy (VC), High-Frequency Ultrasound (HFUS), Reflectance Confocal Microscopy (RCM), Optical Coherence Tomography (OCT), Optical Microangiography (OMAG), Laser Doppler Imaging (LDI), and Multiphoton Tomography (MCT)—Table 3.

**Table 3:** Non-invasive imaging techniques for diagnosis and assessing the response to therapy in psoriasis.

Technique	View/Section	Depth of Penetration	Visualized Structures	Reference
Dermoscopy	Horizontal	-	Superficial vascular pattern	[64]
			Scale distribution	
			Plaque background color	
High-frequency ultrasound (20–100 MHz)	Vertical	1–12 mm	Epidermis and dermis	
Reflectance confocal microscopy	Horizontal	200–300 μm	Epidermis and superficial dermis	
Optical coherence tomography	Horizontal/Vertical	1–2 mm	Epidermis and superficial dermis	
Optical microangiography—an extension of OCT	Vertical/3D	2 mm	Microcirculatory tissue beds	
Laser Doppler imaging	Horizontal	2 mm	Cutaneous blood flow	
Multiphoton tomography—subcellular resolution	Horizontal/Vertical	200–250 μm	Epidermis and superficial dermis	

Dermoscopy, videocapillaroscopy, and videodermoscopy (VD) are non-invasive, in-office techniques that reduce the need to perform biopsies for histopathological examination in patients with psoriasis. In psoriatic skin, standard dermoscopy at a low 10-fold magnification can show the presence of red dots, which represent the top of the tortuous and elongated capillaries within dermal papillae. A higher magnification of 100–400-fold is obtained by VC and VD and highlights the typical “bushy” capillaries with a regular distribution [64,65]. Lallas et al. state that plaque psoriasis was found to correlate significantly with a regular arrangement of dotted vessels, a light red background color, and a diffuse distribution of white scales. This combination of findings allowed for a correct diagnosis with a specificity of 88% and sensitivity of 84.9% [66]. Furthermore, videodermoscopic pictures of psoriatic plaques may differ in the anatomic location, duration of psoriatic plaque, and with patients’ sex, as stated by Golińska et al. in a study evaluating 306 psoriatic plaques. VD with a 20-fold magnification revealed red dots and globules in 100% of plaques arranged in a diffuse pattern (56% of lesions) mainly on the face, patchy lesions on the back (38%), and polygonal lesions on the lower legs (6%). The lesions located on the forearms and scalp, lasting less than or equal to 5 weeks, predominately showed patchy distribution of the scales, whereas older lesions predominately showed diffuse type distribution. Women were also found to more commonly show diffuse distribution of scales in lesions located on the face compared to men [67]. Dermoscopy may also be of aid in predicting therapeutic response of psoriasis vulgaris to NB-UVB, which is affected by the anatomical localization of the lesions. Plaques located on the legs were found to be significantly more resistant than lesions located on other sites. Globular vessels can also be seen as predictor of limited or no clinical response to NB-UVB therapy [68]. Last but not least, dermoscopy might be useful for evaluating the response of psoriatic patients to biological agents. Hemorrhagic dots are an early and favorable predictor of clinical response, while the persistence or recurrence of dotted vessels might predict persistence or subsequent clinical recurrence [69].

High-frequency ultrasound is another useful tool that provides objective measurements in real time, reduces operator bias as compared to clinical evaluation, and is an appropriate technique for assessing plaque thickness, immune-mediated inflammation, and vascularization in psoriasis [70,71]. In psoriatic plaques, HFUS shows a three-layer structure consisting of a hyperechogenic band representing the epidermis with hyperkeratosis and parakeratosis, a subepidermal non/hypoechoic band (SLEB) representing the congested papillary dermis and elongated epidermal rete ridges, and a hyperechogenic band associated with the reticular dermis [72]. In psoriatic onychopathy, one can use Power Doppler mode to see changes such as focal hyperechoic deposits in the ventral plate (subungual keratosis), loss of definition of both nail plates, thickening of the nail bed, and increased blood flow in the nail bed [73].

HFUS appears to be more helpful for monitoring responses to treatment, as previous studies reported. In a pilot study conducted on 30 psoriasis patients treated with Clobetasol propionate 0.05% either once or twice daily, US (20 MHz) showed a reduction of psoriatic skin thickness, with values equal to adjacent healthy skin in all treated plaques [74]. Similarly, Polańska et al. stated that NB-UVB and Calcipotriol/betamethasone dipropionate are equally effective in treating plaque psoriasis patients and that the measurement of SLEB thickness with HFUS is a useful and objective parameter to assess skin lesions [75]. Another study utilizing HFUS (70 MHz) enrolled 10 patients with plaque psoriasis in order to evaluate the lesions before and after 30 days of Ixekiumab treatment. The results showed a rapid action of the drug by decreasing both vascularization and SLEB thickness after 15 days, with a significant reduction in the hyperechoic superficial band after 30 days [76]. Gutierrez et al. compared Power Doppler Sonography (PDS) with clinical and histological findings in 12 psoriasis patients before and after Etanercept treatment, and the results provided a significant correlation between PDS parameters (dermal perfusion) and both the PASI score and histological degree of vascularization [77].

Reflectance confocal microscopy allows the identification of cutaneous cells and tissues up to the papillary dermis with a resolution comparable to conventional histopathology. A near-infrared light beam passes between cellular structures and reflectance occurs at the interface between structures at different refraction indices. The reflected light is subsequently captured and converted into a two-dimensional virtual section which is perfectly matched with clinical and dermoscopic aspects [78,79]. Characteristics of psoriatic plaques under RCM examination include parakeratosis, papillomatosis, acanthosis with a honeycomb pattern of the epidermis, and dilated blood vessels in the epidermis. The thickness at which you observe the tissue using RCM ranges from 75–300 μm compared to normal skin (60–90 μm) [80]. Moreover, Zhong et al. reported that RCM can be used in the identification of Munro’s microabscess in psoriatic plaques with a sensitivity of 90% and specificity of 96.4%, highlighting the role of RCM as a promising tool in the diagnosis and differential diagnosis of psoriasis vulgaris [81]. RCM’s real applicability lies in its ability to view and evaluate in real time the microscopic changes in psoriatic plaques during treatment. In a pilot study conducted on six patients with psoriasis undergoing UVB phototherapy, the results showed a high correlation between clinical, RCM, and immunohistochemical features at time zero before the first irradiation, after nine irradiations, at clearance, and 12 weeks after clearance [82]. RCM is also able to give useful and practical information about follow-up in patients under Adalimumab treatment. The findings showed variations in the confocal parameters that had a significant reduction in infiltration of inflammatory cells in both epidermal and superficial dermal inflammatory cells after 4 weeks of treatment, prior to a clinical reduction in the erythema of lesions [83].

Optical coherence tomography is a non-invasive method using infrared light for morphologic investigation of tissues. Two-dimensional images comparable with virtual biopsies are displayed in real time and represent backscattered signals in a negative grayscale. The scattering characteristics of the skin depend on the distribution of cells, orientation and density of collagen fibers, and melanin content [84,85]. In psoriasis, OCT provides an accurate image of the Stratum Corneum (SC), seen as a hyper-reflective band easily distinguishable from the underlying epidermis. One can also visualize a thickening of the epidermis, stratum granulosum, and spinosum as a honeycomb pattern (polygonal keratinocytes with hypo reflective nuclei). The hypo-reflective dermo–epidermal junction and the hyper-reflective dermis correlated with collagen and elastic fibers, and the dark canalicular structures corresponding to dilated blood vessels can also be seen [86,87]. Ha-Wissel et al. conducted a study with three psoriasis patients undergoing treatment with Ixekizumab, Risankizumab, and Certolizumab and found OCT to be an adequate imaging technique for the objective quantification of epidermal thickness (ET) and vascular parameters (pattern, density, diameter, depth, and count) before and after biological therapy. At baseline, the mean epidermal thickness of psoriatic lesions was 405.8 μm, compared to 90–100 μm in control, perilesional skin. ET decreased during the observation period and was further halved: Ixekizumab (after 3.95 weeks), Certolizumab (after 4.23 weeks), and Risankizumab (after 6.08 weeks). Moreover, there was a positive significant correlation between ET in lesional skin and PASI clinical score. After 52 weeks, full normalization of the vessel count and a decreased vascular density due to a reduction of vessel diameter were observed [88]. In another study, 23 patients were treated with topical corticosteroids, cyclosporine, or phototherapy and evaluated at baseline and 1 and 2 months after treatment. Evaluation was based on ET assessed by OCT, changes in histopathological disease severity, PASI score, and self-reported measures of disease severity such as DLQI score. The results showed higher ET in untreated plaques, measuring 30–40 μm thicker than in normal healthy skin, and a significant decrease in ET in plaques undergoing treatment at visits 2 and 3. Interestingly, ET significantly correlated with self-reported measures of disease but not with the PASI score [89]. OCT may be of aid even in psoriatic nail imaging, offering an objective evaluation and three-dimensional reconstruction of nail structure, pre and post-treatment response image storage, and the capability of measuring nail thickness [90]. Aydin et al. compared OCT and US for nail disease assessment in 18 psoriasis patients and revealed that OCT was able to detect subtle abnormalities in 12 clinically normal nails and in 41 nails with normal US, suggesting that OCT has the potential to become the gold standard for imaging the nail in patients with psoriasis [91].

Although skin biopsy remains one of the widely reported approaches for skin analysis, apart from the imaging techniques previously mentioned, less invasive alternatives have emerged in the dermatological field that allow biomarker quantification in patients with psoriasis. This approach enables faster routes to diagnosis and detection of key pathophysiological mechanisms and allows for an optimized/personalized treatment plan along with measuring response to treatment [92]—Table 4.

**Table 4:** Novel techniques/methods for assessing psoriasis ‘molecular footprint’. (↑ increased, ↓ decreased).

Author, Year	Technique	Outcome	*p*
[93]	Skin surface wash sampling (followed by ELISA quantification of TNF-α, IL-1α, and IL-6 cytokine levels)	TNF-α secretion ↑ in psoriatic lesional skin compared to psoriatic non-lesional area	*p* < 0.05
		IL-1α, IL-6 level ↑ in lesional skin compared to control skin	*p* < 0.01 *p* < 0.05
[94]	Tape stripping and ELISA analysis of S100A8/S100A9 heterodimer	Significant ↑ levels of SC S100A8/A9 in psoriasis lesional skin compared to non-lesional and control skin	*p* < 0.001
		S100A8/A9 levels in SC of psoriasis patients positively correlated with PASI score	*r* = 0.265, *p* = 0.019
[95]	FibroTX Transdermal Analysis Patch (TAP) and ELISA for IL-1α, IL-1RA, CXCL-1/2, hBD-1	IL-1RA, CXCL-1/2, hBD-1 levels ↑ in lesional versus non-lesional psoriatic skin	*p* < 0.001 *p* < 0.01 *p* < 0.05
		IL-1α ↑, CXCL-1/2 ↓ in healthy versus lesional psoriatic skin	*p* < 0.001 *p* < 0.01
		A clear reduction in IL-1RA and CXCL 1/2 on lesional skin after 4w of NB-UVB treatment	*p* < 0.01 *p* < 0.05
		Positive correlation between CXCL-1/2 and SLEB thickness	*r* = 0.512, *p* = 0.004
[96]	Gingival Crevicular Fluid (GCF) and Multiplex bead-based immunoassay for IL-18, sICAM-1, and sE-selectin	IL-18 GCF levels ↑ in psoriatic patients versus controls	*p* < 0.05
		sE-selectin GCF levels ↓ in psoriasis patients versus healthy controls	*p* < 0.05
		Psoriasis was associated with IL-18 and E-selectin levels regardless of periodontal status, age, and smoking	*p* < 0.05
[97]	Saliva samples, ELISA for IL-1β assessment	Significantly ↑ salivary IL-1β levels in patients versus controls at baseline	*p* < 0.0001
		TNF-α inhibitor treatment significantly ↓ IL-1β levels compared with baseline	*p* = 0.0002
		Positive correlation between PASI score and IL-1β level after treatment	*r* = 0.21, *p* = 0.0028

## 3. Pathophysiology

### 3.1. Brief Chronology

**Figure 1:**
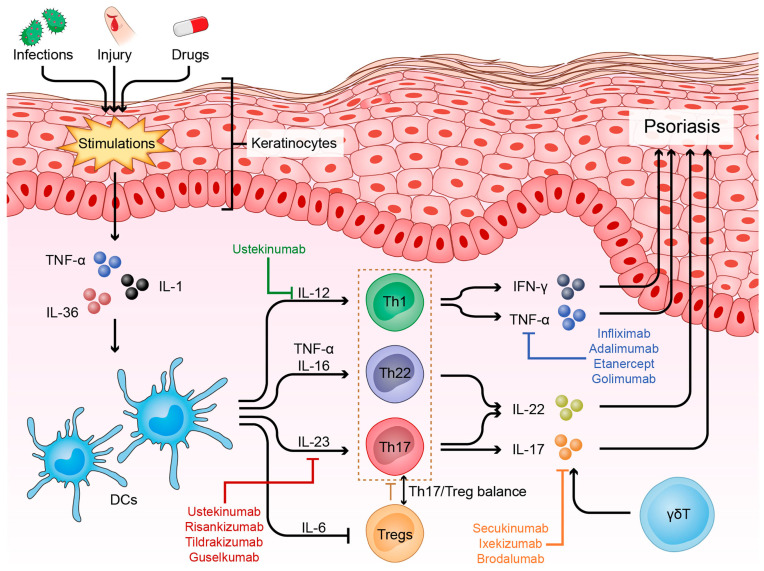
Overview of psoriasis molecular pathogenesis and targeted therapies.

Throughout the 1980–90s, psoriasis was assumed to be an immune-mediated disease, due to researchers identifying cytotoxic T cells around the capillaries, epidermis, and dermis of psoriatic lesions. Subsequently, the characterization of specific T cell populations was carried out based on their secretory cytokine profile. They found that Th1 cells secreted IFNγ and TNF-α and that Th2 cells secreted IL-4, IL-5, and IL-13, thereby defining psoriasis as a Th1 disease [98]. Other predominate disease models showed that IFNγ and IL-12 could be the main drivers of the disease. These data supported in the late 1990s the discovery of cytokines related to IL-12, specifically IL-23, which comprises a specific p19 subunit and a common p40 subunit shared with IL-12. By the early 2000s, it was shown that IL-23 stimulated the production of IL-17A by a subpopulation of T cells negative for both IFNγ and IL-4, thus introducing Th17 cells to the conventional Th1-Th2 classification of autoimmune diseases [99]. Advances in the decoding of the cytokine circuits and molecular and cellular pathways involved in psoriasis led to the development of highly effective targeted therapeutic agents (Figure 1).

### 3.2. Decoding Cytokine Circuits

Based on results from GWAS studies, the IL-23/Th17 axis is considered to be the most important driver of psoriasis [100,101], with several clinical trials revealing the efficacy of current IL-23 and IL-17 inhibitors in use [102].

IL-23 is a proinflammatory cytokine belonging to the IL-12 family, which also comprises other heterodimeric cytokines such as IL-12, IL-27, and IL-35 [103]. IL-23 is mainly produced by DCs and activated macrophages located in barrier sites such as skin, intestinal mucosa, and lungs [104]. The receptor of IL-23 is a heterodimer composed of IL-23R which signals through Jak2 and of IL12Rβ1 signaling through Tyk2. By binding to its receptor, IL-23 activates STAT3, ultimately leading to RORγt expression and the dependent differentiation of naïve T cells into Th17 cells (process elicited by IL-6 and TGF beta released by DCs), highlighting the key role of IL-23 in maintaining the Th17 phenotype [105,106,107]. The pathological outcomes of IL-23 signaling are associated with its ability to stimulate the production of several proinflammatory mediators: IL-17, IL-22, GM-CSF, and TNF-α by CD4+ Th17 cells, Tγδ cells, NKT cells, and Innate Lymphoid Cells 3 (ILC3) [108]. Furthermore, IL-23 plays an important role in bridging innate and adaptative immune responses together with IL-12. Both responses can be triggered immediately by macrophages and DCs after a pathogen insult (with IL-12 driving NK production of IFNγ and IL-23 driving IL-17 production by NK cells and neutrophils) and continue to exert their effects on the IL-23/Th17 pathway and Th1 pathway, respectively, during an autoimmune response [109].

The IL-17 family is formed by six members/isoforms (IL-17A-IL-17F), with IL-17A and IL-17F protein levels increased by 6.7-fold and 8-fold, respectively, in psoriatic skin compared to non-lesional skin. IL-17A and IL-17F share 50% identity and bind to the same heterodimeric receptor formed by the IL-17RA and IL-17RC subunits [110]. Once the bond between the IL-17A homodimer, IL-17A-IL-17F heterodimer, or IL-17F homodimer is established, the Act1 cytoplasmatic protein is recruited to the complex (at the C-terminus SAFIR domain) [111] and leads to the recruitment/activation of other signaling molecules such as TRAF6, TRAF2, and TRAF5. Ultimately, TRAF 6 stimulates the activation and phosphorylation of IᴋB kinase through its proteasomal degradation, and to NF-ᴋB release [112]. Th17 (CD4+) cells are the main source for IL-17. However, this cytokine can also be produced by CD8+ T cells, ILCs, γδTcells, neutrophils, mast cells, and macrophages [113,114]. IL-17A exerts its pathological effects through interaction with epidermal keratinocytes by stimulating them to produce IL-19, which in turn promotes keratinocyte migration and activates fibroblasts to produce Keratinocyte Growth Factor (KGF) [115]. Additionally, IL-17A upregulates the expression of CXCL1 and CXCL8 (IL-8) chemokines, which are potent chemoattractants for neutrophilic recruitment [116,117] and CCL20 production. The succeeding interaction between CCL20 and its receptor, CCR6, which is expressed on various IL-17-producing cells, is responsible for maintaining the inflammatory cycle in psoriatic plaques [118]. Last but not least, Th17 cytokines act on keratinocytes to promote the production of antimicrobial peptides: β defensins and S100A7–9, which also contribute to sustaining the inflammatory response and maintaining epidermal hyperplasia [119].

TNF-α is another proinflammatory cytokine initially released by stressed keratinocytes. Its secretion stimulates DC activation and IL-12 secretion, the latter inducing the differentiation of naïve T cells into the Th1 phenotype. Thereafter, Th1 cells produce large amounts of IFN-γ and TNF-α, leading to the perpetuation of TNF-α DCs’ mediated activation [120]. Concretely, TNF-α promotes keratinocyte proliferation and secretion of other inflammatory interleukins, specifically IL-1, IL-6, IL-8, NF-ᴋB, and neutrophilic chemoattractants. It also promotes the proliferation of several adhesion molecules, such as ICAM-1, P, and I-selectins, which facilitate the influx of inflammatory cells in psoriatic skin [121,122].

TNF-α and IL-17A act in a synergic manner to maintain the cytokine cascade in psoriasis. TNF-α stabilizes IL-17A mRNA and increases the expression of IL-17AR in keratinocytes, whereas IL-17A induces TNFR expression [123].

In a classical model of interaction between Antigen Presenting Cells (APCs) and T cells, activated macrophages and DCs release IL-12, which stimulates NK cells to produce IFN-γ and directs naïve CD4+ T cells to Th1 differentiation and the subsequent secretion of IFN-γ. The IFN-γ then enhances the antigen presentation capability of macrophages and DCs. Besides NK and CD4+ cells, there are several other sources of IFN-γ: CD8+T cells, γδTcells, B cells, and even APCs (via IL-12 production in an autocrine manner) [124]. Prior to the IL-23/Th17 driving psoriasis era, the IL-12/IFN-γ signaling pathway was considered the main actor in the pathogenesis of psoriasis. Then, researchers discovered the role of IFN-γ in relationship with the IL-23/Th17 axis, leading to the discovery of IL17/IFNγ-producing T cell subsets (Th17.1) [125]. This subset of T cells expressing both CCR6 and CXCR3 may be of aid when monitoring anti-IL17 treatment response, as Tsiogkas et al. reported a sharp decrease of Th17.1 in the peripheral blood of psoriatic patients undergoing Secukinumab or Brodalumab treatment [126]. IFN-γ may also play a role in driving IL-23 and IL-1β production from myeloid dendritic cells (mDCs) and in stimulating IL-17 production by memory T lymphocytes [127].

The IL-36 family is formed by three agonists (IL-36α, β, γ) and one antagonist (IL36Ra), one accessory protein (IL-1RAcP), and a receptor-IL-36R. IL-36 agonists bind to IL-36R, resulting in a complex that recruits IL-1RAcP, leading to the unification of intracellular TIR domains of receptors, leading to NF-ᴋB and MAPK signaling [128]. The IL-36 receptor is expressed by epithelial cells and antigen-presenting cells, but not by T cells or neutrophils [123]. IL-36 cytokines require different enzymes for proteolytic processing at the N-terminus for activation, and thus *Streptococcus pyogenes* and fungal pathogens increase the expression of IL-36 in epithelial tissues due to pathogen-derived proteases that can process these IL-36 agonists [129]. The enhanced processing of IL-36 agonists contributes to increased IL-36 activation reported in GPP. Immunohistochemistry analysis detected IL-36α and IL-36γ expression by both Psoriasis Vulgaris (PV) and GPP keratinocytes, but with highly intense staining in these keratinocytes disposed around the pustules, highlighting the role of IL-36 in promoting neutrophilic responses and sterile pustules formation, respectively [130]. IL-36γ may serve as a biomarker for psoriasis diagnosis, as *IL-36G* gene expression and immunohistochemical evaluation of IL-36γ in psoriasis vulgaris skin samples were overexpressed. Furthermore, IL-36γ serum levels have proven useful in the monitoring of treatment response, correlating with disease activity scores before and after TNF-α treatment [131].

## 4. Psoriasis Comorbidities

**Figure 2:**
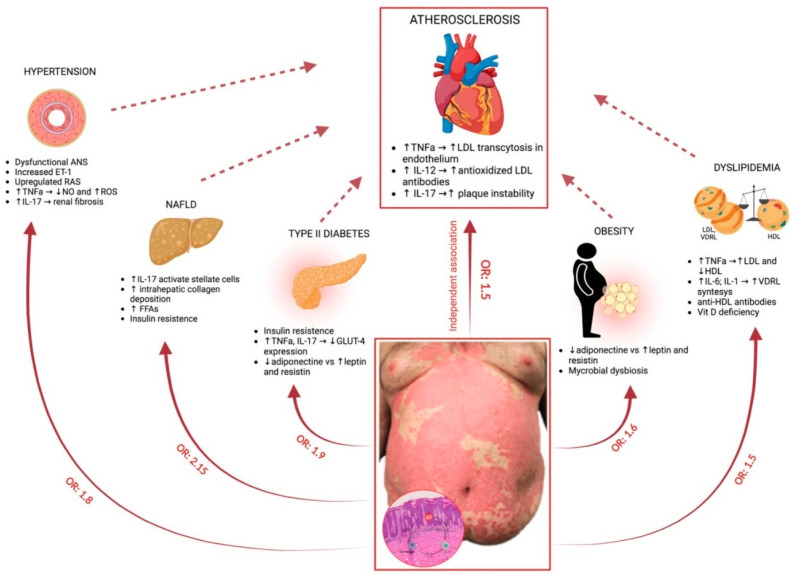
The molecular pathways involved in the link between psoriasis and its comorbidities. Continuous red arrows show the Odds Ratio (OR) for developing different comorbidities in a patient with psoriasis; discontinuous red arrows connect all these conditions with atherosclerosis (↑ increased, ↓ decreased).

Inflammatory mediators and cytokines can circulate in the whole body, thereby deeming psoriasis as a systemic inflammatory state that drives organ dysfunctions and successively attracts different comorbidities (Figure 2). Recognition and adequate treatment of these comorbid conditions are important aspects to consider when managing psoriasis patients, as they can improve their lifespan and quality of life. In a meta-analysis conducted by Tang et al., psoriasis showed a greater risk of organ-based comorbidities compared to the general population. The Proportional Reporting Ratio (*pRR*) was 1.20 (95% CI 1.11–1.30) for cardiovascular disease and 1.56 and 1.75 for cerebrovascular and liver diseases, respectively. Additionally, psoriasis had a higher risk for multiple organ-based comorbidities, including synchronous renal and cerebrovascular events, renal and liver disease, and cardiovascular and liver diseases [132].

### 4.1. Psoriasis and Cardiovascular Disease

Several studies have demonstrated that psoriasis is associated with vascular inflammation and Cardiovascular Disease (CVD) events, mainly Major Adverse Cardiovascular Events (MACE) such as stroke, myocardial infarction, and CVD death. Psoriasis is an independent risk factor for myocardial infarction in both sexes in adults, and the incidence of atherosclerosis is higher in patients with psoriasis compared with controls [133]. Considering this, psoriasis was included in the European Guidelines on cardiovascular prevention as a 1.5x risk multiplier for CVD risk depending on disease severity and with recommendations of statin treatment association [134]. Furthermore, the risk of CVD is correlated with the severity of psoriasis. A study evaluated 115 patients with psoriasis using Fluorodeoxyglucose-Positron Emission Tomography/Computer Tomography (FDG-PET/CT) to assess vascular inflammation and showed that psoriasis severity was associated with the degree of vascular inflammation and an 11% reduction in aortic vascular inflammation coincided with a 75% reduction in the psoriasis severity index [135]. Cumulative exposure to chronic psoriasis-driven inflammation increases the risk of developing MACE, with a one percent chance per year of psoriasis duration [136]. Hjuler et al. stated that biological therapy with anti-TNF agents (Adalimumab, Etanercept, Infliximab) or anti-IL12/23 (Ustekinumab) in patients with severe psoriasis decreases systemic inflammation, thus preventing coronary artery disease progression. Results were assessed via coronary CT angiography before and after 13 months of treatment and showed that Coronary Artery Calcium Score (CAC) scores remained stable, while the severity of luminal narrowing in the diseased segments was unchanged in the intervention group compared to the control group [137]. In another study, Wu et al. observed that the MACE Hazard Ratio (HR) was 45% lower in patients with psoriasis undergoing TNF inhibitor therapy compared to the conventional Methotrexate treatment group after 12 months, therefore highlighting the beneficial role of anti-TNF use as a therapeutic agent [138]. IL-17R is expressed in endothelial cells and once activated leads to the production of TNF-α, IL-1β, CCL20, and ICAM-1, causing endothelial dysfunction [139]. Moreover, IL-17 might enhance oxidative stress by activating NADPH oxidase in vascular smooth muscle cells and through superoxide production via the recruitment of inflammatory cells. In line with this, IL-17 inhibitors showed their ability to reduce the burden of coronary plaques by reducing necrotic core when assessed by CT angiography and compared to other biologic treatment options (anti-IL12/23 or TNF inhibitors) [140]. Different subtypes of T cells, macrophages, and neutrophils might explain the pathophysiological link between psoriasis and atherosclerosis (ATS). In patients with unstable angina and Acute Coronary Syndrome (ACS), Th1, Th17 cells, and IL-17 were found to be elevated when compared to those with stable angina [141]. T reg function was also decreased in both psoriasis and CVD individuals. In patients with psoriasis, it was further seen that macrophages secreted microparticles (MPs) which promoted coagulation disorders, affecting the thickness of intima and media in blood vessels, thus promoting atherosclerosis. Lastly, a new subtype of neutrophils Low-Density Granulocyte (LDG) mediated the production of type I IFNs, possessing an increased ability to form Neutrophil Extracellular Traps (NETs) spontaneously, and was highly abundant in ATS plaques and in the plasma of patients with psoriasis [142].

### 4.2. Metabolic Syndrome (MetS)

**Figure 3:**
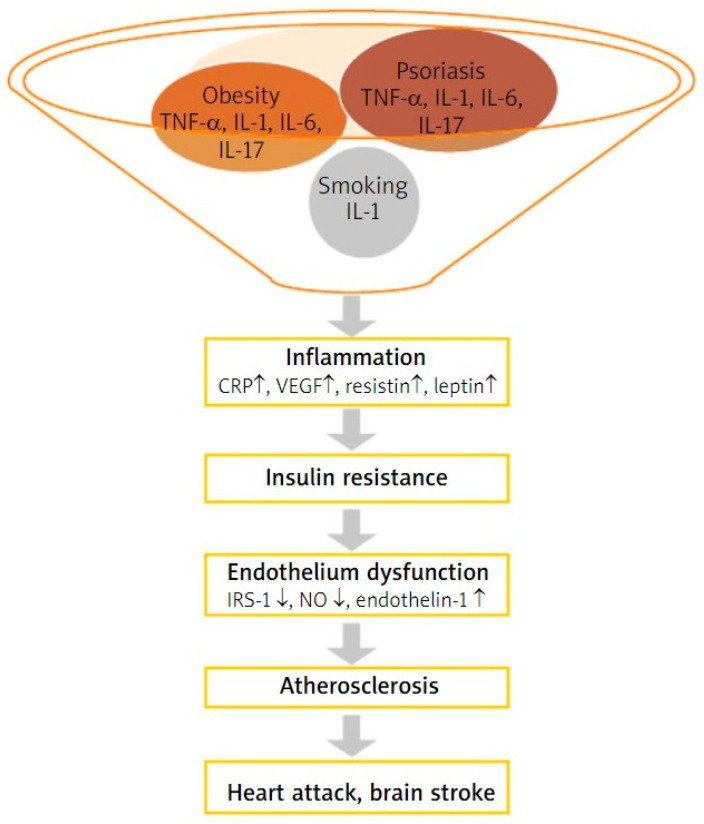
Psoriatic march. Continuous gray arrows show the chronology of events ultimately leading to heart attack or brain stroke (↑ increased, ↓ decreased).

According to National Cholesterol Education Program Adult Treatment Panel III guidelines (NCEAP ATP III), metabolic syndrome is defined as the presence of three characteristics from the following five: central adiposity (waist circumference in men > 102 cm and in women > 88 cm), hypertriglyceridemia > 150 mg/dL, low HDL cholesterol (<40 mg/dL in men and <50 mg/dL in women), Blood Pressure (BP) higher or equal to 130/85 mmHg, and fasting plasma glucose > 100 mg/dL or drug treatment for Diabetes Mellitus (DM) [143]. The prevalence of MetS in patients with psoriasis ranges from 20% to 50%, with a significant association between PASI score and the prevalence of MetS [144]. From a genetical point of view, the overlap between psoriasis and MetS may be explained by *IL-12B*, *IL-23R*, and *IL-23A* gene variants related to psoriasis susceptibility but also to risk for developing type II DM. GWAS studies also revealed that dyslipidemia, hypertension, and CVD-related genes were linked to increased risk of psoriasis [145]. Adipocytokines play an important role in linking psoriasis and MetS. Low adiponectin levels in patients with psoriasis are associated with increased TNF-α and IL-6 levels and are inversely correlated with PASI scores. Increased leptin levels were observed in obese psoriasis patients compared to obese patients [146], while serum and lesional skin levels of leptin and leptin receptor expression were higher in patients (all with normal BMI) with greater severity of psoriasis when compared to less severe disease and controls, showing a positive correlation with disease duration [147]. It is known that obesity promotes production of IL-17 from T cells in visceral fat and peripheral tissues, and that high IL-17R levels correlate with Insulin Resistance (IR) while an IL-17 blockade decreases hepatic inflammation in Non-Alcoholic Steatohepatitis (NASH) syndrome [148]. Conversely, the overexpressed IL-17 cytokine in psoriasis leads to the activation of the NF-ᴋB pathway and the production of other proinflammatory cytokines-IL-1β, IL-6, and TNF-α [149]. TNF-α contributes to IR by inhibiting the expression of GLUT4 (an insulin-dependent transporter localized in adipocytes, skeletal, and cardiac muscles) and by inducing the phosphorylation of Insulin Receptor Substrate-1 (IRS-1), thus inhibiting insulin signaling [150]. In a study conducted by Fatima et al., type II diabetes patients expressed a significantly higher serum level of IL-1β, IL-6, IL-17A, and IL-22 levels compared to controls, suggesting that besides the involvement of Th1 and Th2 cells, Th17 cells might be involved in type II diabetes [151] pathogenesis as in psoriasis. Obesity is also correlated with increased levels of circulating macrophage Migration Inhibitory Factor (MIF) as a result of adipocyte expansion and adipose tissue inflammation. MIF regulates inflammation through the recruitment of various inflammatory cells via CXCR2 and CXCR4 signaling and through its tautomerase and oxidoreductase activity by modulating insulin release as it co-localizes with insulin in secretory granules of pancreatic cells [152]. On the other hand, MIF promotes the uncontrolled proliferation of keratinocytes in murine models of psoriasiform dermatitis, inflammatory cell infiltration with monocyte-derived cells, and dermal angiogenesis [153], suggesting its role as a disease promoter in psoriasis and as a linking molecule between psoriasis, obesity, and IR. The close relationship between metabolic syndrome and psoriasis due to their similar pathophysiological mechanism leads to the concept of psoriatic march, in which proinflammatory cytokines with adipokines can cause IR, endothelial dysfunction, and subsequent atherosclerotic plaque formation (Figure 3).

### 4.3. Inflammatory Bowel Disease (IBD)

Psoriasis and IBD may coexist, as they share common predisposition genes and immunological pathways. The bidirectional Mendelian randomization analysis highlights the evidence for bidirectional dual causality between psoriasis and Chron Disease (CD). It finds that the presence of IBD is responsible for the development of psoriasis, including psoriatic arthritis, and that psoriasis and psoriatic arthritis increased the odds of developing CD [154,155]. From a genetic perspective, GWAS studies identified 11 genes of predisposition overlapping in psoriasis and IBD. Furthermore, CD and psoriasis share seven non-HLA susceptibility loci [156], and the most important susceptibility locus for psoriasis is located on chromosome 6-p21, which is very close to the one implicated in CD pathogenesis (IBD-3 p23) and to the TNF-α encoding gene [157]. Immunologically speaking, psoriasis may coexist with IBD, establish a bidirectional causative relationship, or can be a paradoxical adverse event of anti-TNF therapy. The immunological links between psoriasis and IBD are linked by defects in the structure and function of the intestinal and epidermal barrier, augmenting the interaction of antigens with DCs. This results in DC activation; NK cell production of IFN-γ, TNF-α, and IL-23; and degranulation of connective tissue mast cells in the intestine and human skin, creating an ideal microenvironment for neutrophils and lymphocytes [158]. Interestingly, anti-TNF-α agents used for IBD treatment can induce paradoxical psoriasis due to the hyperactivation of pDCs and their secretion of IFN-γ (IFN secretion is generally inhibited by TNF-α). In line with this, IL-17 antibodies used for psoriasis treatment may trigger IBD or worsen bowel inflammation because IL-17 neutralization affects tissue homeostasis and the repairing process and also impairs intestinal wall integrity. As IL-23 antibodies inhibit solely the production of IL-17 from Th17 cells, other cellular sources of IL-17 remain unaffected, therefore highlighting their beneficial role in the treatment of concomitant psoriasis and IBD [159]. The gut microbiota is increasingly recognized as an important environmental factor for the development of IBD and even psoriasis. IBD and psoriasis patients bear an altered intestinal microbiome characterized by a decrease in *Faecalibacterium Prausnitzii*, together with an increase in *Escherichia coli*. *Faecalibacterium Prausnitzii* is able to secrete anti-inflammatory molecules that modulate the host immune system and is one of the most abundant sources of butyrate and also an inducer of T regs. Thus, it is involved in maintaining the balance between T effector/T reg cells. These results support the presence and importance of a gut-microbiome–skin axis [160].

### 4.4. Other Comorbidities: Autoimmune Skin Disorders Linked to Psoriasis

There are several other conditions that occur more frequently in patients with psoriasis than in the general population that have been reported by scientists, such as sleep disorders, thyroid autoimmune disease, lung fibrosis, osteoporosis, and Nonalcoholic Fatty Liver Disease (NAFLD). The potential physiopathological mechanisms that could explain their correlation with psoriasis are listed in Table 5.

**Table 5:** Other comorbid conditions in psoriasis.

Comorbid Condition	Physiopathological Mechanisms	Reference
Sleep disorders	Lower melatonin levels in patients with psoriasis	[161]
	Increased levels of TNF-α and IL-6 in psoriasis and in cases of sleep restriction	
	Role of substance P in keratinocyte proliferation and in sleep disorders	
Thyroid autoimmunity	Th1 immune response in psoriasis, PsA, and Autoimmune Thyroiditis (AT), high circulating levels of CXCL10	[162]
	Th17 percentage positively correlated with serum TPOAb, TgAb, and TSH levels in patients with Hashimoto thyroiditis	[163]
	Disturbed NF-ᴋB signaling in both AT and psoriasis	[164]
Lung disease	In murine lung epithelium, IL-17 led to a Chronic Obstructive Pulmonary Disease (COPD) pattern of inflammation	[165]
	Treatment with anti-IL-23 antibody reduces fibrosis, IL-17A and IL-22 levels in a murine model of pulmonary fibrosis exacerbation	
	The severity of psoriasis determines the risk of COPD	[166]
Osteoporosis	TNF-α and IL-6 increase the production of Receptor Activator of NF-ᴋB Ligand (RANKL) and osteoprotegerin, which stimulate osteoclastogenesis	[167]
NAFLD	TNF-α, IL-6, and IL-17 may contribute to psoriatic plaque development, impaired glucose metabolism and IR in hepatocytes and adipocytes	[168]

Consistent with its autoimmune nature, psoriasis has been linked to other autoimmune skin disorders. Several intriguing links to vitiligo, Hidradenitis Suppurativa (HS), and Bullous Pemphigoid (BP) have been observed.

In both psoriasis and vitiligo, Th1 cells play a central role in pathogenesis [169]. Moreover, Kun-Ju Zhu et al. found through multiple regression analysis of SNPs in MHC loci that rs 9468925 in HLA-C/HLA-B is shared by psoriasis and vitiligo [170].

Contrastingly, when BP is present along with psoriasis, no common susceptibility HLA alleles were reported to coincide, although they share some important physiopathological mechanisms. The degradation of laminin in psoriasis causes modifications in the basement membrane, which leads to the development of anti-Basement Membrane Zone (BMZ) antibodies. IL-1β promoted skin inflammation in a mouse model of BP, while in psoriasis, IL-1 interferes with adhesion, proliferation, and epidermal differentiation, therefore contributing to plaque formation [171].

Regarding patients suffering from both psoriasis and HS, the IL-23/Th17 axis was found to be the common denominator, as experimental studies stated that IL-23 is overexpressed by macrophages in lesions of HS in addition to CD4+ T cells producing IL-17 [172].

Although further studies are necessary for establishing the real relationship between these conditions and psoriasis, there is no question that the inflammatory cascade in psoriasis is incriminated in the morbidity burden for these patients and it is likely that a holistic management would aid clinical outcomes.

## 5. Therapeutical Approach

Treatment approaches for plaque psoriasis should take into consideration disease severity and the association of several medical conditions because they can impact the clinical decisions and treatment plans [173,174].

Developing a consensus statement on the classification of psoriasis severity to aid treatment decisions led the International Psoriasis Council to propose a statement that rejects mild, moderate, and severe categories in favor of a dichotomous definition. Their definition states that patients should be classified as candidates for topical or systemic therapy, with the latter meeting at least one of the following criteria: Body Surface Area (BSA) >10%, disease involving special areas, and failed response to topical therapy [175].

As biologic treatment has emerged, there is an increasing effort to adopt a treat-to-target approach for psoriasis. One must have a PASI score less than or equal to two and a clear/almost clear Physician’s Global Assessment (PGA), which represent a tangible and realistic endpoint [176].

### 5.1. Biologics

There are currently 11 biologics in four different classes (anti-TNF-α, anti-IL12/23, anti-IL17, and anti-IL23p19) approved by the FDA for psoriasis treatment. Biologic agents interact with a specific cytokine, exerting their effect via targeted immunomodulation, being both safe and efficient in treating psoriasis in comparison to traditional immunosuppressant drugs [177].

TNF-α inhibitors currently in use to treat psoriasis are Etanercept, Infliximab, Adalimumab, and Certolizumab. Etanercept is a dimeric fusion protein of tumor necrosis factor receptor 2 (or p75) and the Fc portion of human IgG1, which prolongs the half-life of the active substance in the bloodstream [178]. Except for Etanercept, all anti-TNF-α agents are monoclonal antibodies. Infliximab is a chimeric monoclonal antibody consisting of the IgG1ᴋ human constant region (75%) and murine variable region (25%), binding to both soluble and transmembrane forms of TNF-α and thus disrupting the interaction of TNF-α with its receptors [179,180]. Adalimumab is a fully human antibody that blocks TNF-α interaction with the p55 and p75 cell surface TNF receptors [181]. Certolizumab pegol is a humanized antigen fragment (Fab’) of a monoclonal antibody that has been conjugated with polyethylene glycol. Certolizumab lacks an Fc region, a fact that minimizes the Fc-mediated effects of complement-dependent cytotoxicity or antibody-dependent cell-mediated cytotoxicity [182]. The biological consequence of PEGylation is enhanced retention of protein conjugates in the blood and reduced immunogenicity [183]. Furthermore, Certolizumab pegol treatment may be continued during pregnancy when considered necessary to control disease activity, as it does not cross the placenta [184].

Ustekinumab is a fully human, IgG1ᴋ monoclonal antibody that binds the shared p40 subunit of IL12 and IL23 [185], but this inhibition of IL12 signaling may be counterproductive. This is because IL12 plays a regulatory role by lowering the invasion of γδT17 cell subset and by initiating a transcriptional program in keratinocytes that limits skin inflammation [186].

Guselkumab, Tildrakizumab, and Risankizumab target IL-23 by inhibiting the p19 subunit without disrupting the IL-12 signaling pathway with a fourth anti-IL-23p19 agent, Mirikizumab, showing favorable safety profiles and efficacy levels at week 16 and 52 compared to placebo in phase III clinical trials [187]. These antibodies targeting IL-23 display different characteristics regarding receptor affinity, in vitro potency, and in vivo efficacy, as demonstrated by Zhou et al. in a comparative study. They showed that Risankizumab and Guselkumab had a five-fold higher affinity for the receptor and completely blocked the binding of IL-23 to IL-23Rα as compared to Tildrakizumab. Furthermore, Risankizumab and Guselkumab had a more prominent effect on IL-17, IL-22, and keratinocyte gene expression reduction [188]. Another study measured the concentration of human recombinant IL-23 in cynomolgus monkeys and found that the predicted rank order of reduction of free IL-23 was in concordance with the reported rank order of PASI 100 scores in clinical trials, with Risankizumab showing the highest degree of target suppression, followed by Guselkumab and Tildrakizumab [189].

Three monoclonal antibodies against IL-17 have been approved: Secukinumab, Ixekizumab, and Brodalumab. Both Secukimuab and Ixekizumab target IL-17A [190], while Brodalumab binds with high affinity to the IL17RA subunit, simultaneously inhibiting IL-17A, IL-17F, IL-17C, and IL-17E and providing a > 95% improvement in upregulated psoriasis genes after 12 weeks of treatment [191]. Bimekizumab, a novel humanized monoclonal IgG1 antibody that neutralizes both IL-17A and IL-17F, has been recently approved for the treatment of moderate to severe psoriasis in adults, promising a PASI 90 and PASI 100 score in 4 weeks and maintenance of effectiveness up to 52 weeks, with a good safety profile [192].

### 5.2. Small Molecule Therapies

Small molecules are small-molecular-weight inhibitors (<1 kDa) that can enter the cells and selectively inhibit signaling pathways [193]. Some advantages they offer to traditional therapies are the topical or oral route of administration, lack of immunogenicity, and low costs of production [194].

Apremilast is an oral PDE4 inhibitor that leads to an increase in intracellular cAMP level, the subsequent production of the anti-inflammatory cytokine IL-10, and the decreased production of pro-inflammatory TNF-α, IL-23, and IFN-γ [195,196]. Another oral PDE4 inhibitor, Orismilast IR (instant release), proved its efficacy and safety for moderate to severe psoriasis in a phase 2a trial with 44.4% of patients achieving PASI 75 response compared to the placebo group (5.6%) at week 16 [197]. Topical PDE4 inhibitor- Roflumilast was approved by the FDA in July 2022 for the treatment of plaque psoriasis for adults and children over the age of 12 [198].

Deucravacitinib is a highly selective TYK inhibitor oral drug first approved in the USA in September 2022 for moderate to severe plaque psoriasis and immediately thereafter in Japan for two other clinical forms of psoriasis—GPP and erythrodermic. As IL-12, IL-23, and type I IFN signal via TYK2, Deucravacitinib inhibits the receptor-mediated activation of TYK2, preventing downstream signaling [199]. Baricitinib is the first generation of Jak1/2 inhibitor which showed in a randomized clinical phase 2 trial a 75% reduction in PASI score after 12 weeks, with a dose of either 8 mg or 10 mg once daily compared to placebo, with no serious adverse events [200]. Tofacitinib, an oral Jak1/3 inhibitor currently approved for psoriatic arthritis, has been intensively studied in clinical trials. In phase III trials, a significant proportion of patients receiving Tofacitinib obtained PASI 75 at week 16 [201] and sustained efficacy through 24 months, with 10 mg twice daily providing greater efficacy than 5 mg BID [202].

### 5.3. Nanotechnology-Based Therapies

Psoriatic skin acts as a strong barrier to topical drug penetration due to keratinocyte hyperproliferation. Thereby, nanocarriers can enhance the efficacy of the loaded drug/active substance by increasing its penetration, preventing its degradation, promoting sustained release, and limiting interactions with non-target sites [203]. Advanced nanosystems include metallic, lipidic, polymeric, and hybrid nanocarriers which incorporate different drugs and have been investigated to a greater extent on animal models or cell lines mimicking psoriatic skin [204]. Some of these nanocarriers, their characteristics, and the results based on their utilization in psoriasis are described in Table 6.

**Table 6:** Recent approaches utilizing nanotechnology in the management of psoriasis.

Nanocarrier	Characteristics	Incorporated Drug	Outcome/Results	Study Reference
Nanostructured lipid carrier (NLC)	Solid lipid and oil, resulting in a less ordered lipid matrix which improves the loading and bioavailability of hydrophobic drugs and prevents drug leaching and oxidation [205]	Tacrolimus (TAC) and siRNA against TNF-α	Seven-fold reduction in TNF-α expression after NLC TAC TNF-α siRNA	[206]
Nanoemulsion (NE)	A system consisting of two immiscible phases—oil and aqueous phases—stabilized by surfactants with 20–400 nm droplet size, showing high solubilization capacity for both hydrophilic and hydrophobic actives [196]	Clobetasol Propionate (CP) and Calcipotriol (CT)	Six-fold increase in the duration of drug release for the NE compared to free drug Maximum reduction in skin inflammation and scaly lesions	[207]
			95% and 77.77% reduction in IL-6 and TNF-α serum levels, respectively, after treatment with NE	
Fusogenic nucleic acid-lipid particles (F-NALPs)	Lipid-Based Nanoparticles (LBNPs) with fusogenic lipids can induce lipid fusion between the plasma membrane and cells, promoting cellular uptake of nucleic acids and endosomal escape to targeted genes [198]	STAT3 and TNF-α siRNA	F-NALPs containing siSTAT3 and siTNFα significantly reduced the expression of STAT3 and TNFα mRNA expression	[208]
			Levels of IL-17 and IL-23 shifted back to normal after STAT3 and TNF-α blockade	
Gold NPs (AuNPs)	Metallic NPs with low toxicity, with a large surface area, small size, and anti-inflammatory effect [209]	Methotrexate (MTX)	MTX-conjugated AuNP exhibited a penetration either in the epidermis or dermis and had no toxicity on keratinocytes	[210]

## 6. Future Perspectives, Unsolved Questions, and the Pathway towards Personalized Medicine

Despite the various therapeutic collections that have emerged recently for the treatment of psoriasis, the choice of treatment is still based on empirical criteria like clinical factors and not on the genetic and immunological profile of each patient. Furthermore, some patients might not respond to biologics at all (primary failure) or might subsequently lose response (secondary failure), most likely due to the development of anti-drug antibodies. This drawback might be overcome by measuring the level of drug in the serum during the initial phase of treatment, but this is still a trial-and-error strategy rather than a personalized medicine approach. Hence, the introduction and application of -omics technologies in daily clinical practice may be of aid in identifying the accurate biomarkers that are able to predict which therapy is suitable for each patient, their therapeutic outcomes, and the probability of developing psoriasis-related comorbidities. This approach would allow an early intervention that could modify the disease course, improve patients’ quality of life, and reduce health costs.

Serum cytokines may be an important biomarker in quantifying psoriasis activity and guiding treatment strategy as they correlate with skin cytokines. Studies supporting this reported that TNF-α, IFN-γ, IL-22, IL-6, fibrinogen, and C3 serum levels were higher in psoriasis patients compared with healthy controls (*p* < 0.05), thus making these biomarkers suitable for predicting psoriasis occurrence [211]. Moreover, Olejniczak-Strauch et al. assessed the influence of anti-TNF-α and anti-IL12/23 biologic therapies on the circulating levels of IL-6 and IL-22. They found a statistically significant decrease of IL-6 levels regardless of treatment and of IL-22 levels in patients treated with either Adalimumab or Infliximab. No correlation with PASI score was observed [212]. Likewise, Wang et al. profiled plasma proteins from patients with moderate-to-severe psoriasis to explore potential biomarkers for effective systemic treatment and their potential relationship with CVD. He proposed circulating levels of IL-17C and Peptidase Inhibitor 3 (PI3) as indicators of biological treatment success regardless of the class (anti-TNF-α, IL12/23, IL-17, and IL-23 agents) and IL-17A as a marker of CVD (lower plasma IL-17A levels were associated with increased evidence of CVD) [213].

To date, several studies have been conducted to identify potential biomarkers for tailored biological therapy, but no suitable panel for use in daily routines has been identified yet. However, we might be closer than we think, as Mindera Health validated a test that predicts patients’ responses to all biological drug classes with high positive predictive value (91%), sensitivity (79%), and specificity (86%) using a machine learning method that combines baseline transcriptome data with clinical outcomes as defined by week 12 PASI score [214]. Currently, a clinical trial (NCT05036889) is being conducted to evaluate the Mind.Px machine learning algorithm on response to biologic treatment as judged by PASI score from baseline to the end of week 16 of the study compared to a no-intervention study group where treatment is offered as usual.

## 7. Conclusions

After a thorough review of the literature, we suggest that the intricacies of psoriasis are slowly being revealed. There is a worldwide agreement that the burden of psoriasis is immense, as it has devastating systemic consequences and a cure is unlikely to be identified in the near future despite the efforts of scientists. However, a consensus is emerging on an approach to better match the clinical presentations with the genetic and immunological profile of each patient, allowing doctors to develop tailored treatment plans. While this may sound like something of the future, it is closer to becoming a reality than we think.

## Data Availability

Not applicable.

## References

[1] Samotij D., Nedoszytko B., Bartosińska J., Batycka-Baran A., Czajkowski R., Dobrucki I.T., Dobrucki L.W., Górecka-Sokołowska M., Janaszak-Jasienicka A., Krasowska D. (2020). Pathogenesis of Psoriasis in the “Omic” Era. Part I. Epidemiology, Clinical Manifestation, Immunological and Neuroendocrine Disturbances. Postep. Dermatol. Allergol..

[2] Rapp S.R., Feldman S.R., Exum M.L., Fleischer A.B., Reboussin D.M. (1999). Psoriasis Causes as Much Disability as Other Major Medical Diseases. J. Am. Acad. Dermatol..

[3] Parisi R., Symmons D.P.M., Griffiths C.E.M., Ashcroft D.M., Identification and Management of Psoriasis and Associated ComorbidiTy (IMPACT) project team (2013). Global Epidemiology of Psoriasis: A Systematic Review of Incidence and Prevalence. J. Investig. Dermatol..

[4] Parisi R., Iskandar I.Y.K., Kontopantelis E., Augustin M., Griffiths C.E.M., Ashcroft D.M., Global Psoriasis Atlas (2020). National, Regional, and Worldwide Epidemiology of Psoriasis: Systematic Analysis and Modelling Study. BMJ.

[5] Iskandar I.Y.K., Parisi R., Griffiths C.E.M., Ashcroft D.M., Global Psoriasis Atlas (2021). Systematic Review Examining Changes over Time and Variation in the Incidence and Prevalence of Psoriasis by Age and Gender. Br. J. Dermatol..

[6] Hägg D., Eriksson M., Sundström A., Schmitt-Egenolf M. (2013). The Higher Proportion of Men with Psoriasis Treated with Biologics May Be Explained by More Severe Disease in Men. PLoS ONE.

[7] Dand N., Mahil S.K., Capon F., Smith C.H., Simpson M.A., Barker J.N. (2020). Psoriasis and Genetics. Acta Derm.-Venereol..

[8] Chandra A., Ray A., Senapati S., Chatterjee R. (2015). Genetic and Epigenetic Basis of Psoriasis Pathogenesis. Mol. Immunol..

[9] Tsoi L.C., Stuart P.E., Tian C., Gudjonsson J.E., Das S., Zawistowski M., Ellinghaus E., Barker J.N., Chandran V., Dand N. (2017). Large Scale Meta-Analysis Characterizes Genetic Architecture for Common Psoriasis Associated Variants. Nat. Commun..

[10] Strange A., Capon F., Spencer C.C.A., Knight J., Weale M.E., Allen M.H., Barton A., Band G., Bellenguez C., Genetic Analysis of Psoriasis Consortium & the Wellcome Trust Case Control Consortium 2 (2010). A Genome-Wide Association Study Identifies New Psoriasis Susceptibility Loci and an Interaction between HLA-C and ERAP1. Nat. Genet..

[11] Dand N., Duckworth M., Baudry D., Russell A., Curtis C.J., Lee S.H., Evans I., Mason K.J., Alsharqi A., Becher G. (2019). HLA-C*06:02 Genotype Is a Predictive Biomarker of Biologic Treatment Response in Psoriasis. J. Allergy Clin. Immunol..

[12] Burlando M., Russo R., Clapasson A., Carmisciano L., Stecca A., Cozzani E., Parodi A. (2020). The HLA-Cw6 Dilemma: Is It Really an Outcome Predictor in Psoriasis Patients under Biologic Therapy? A Monocentric Retrospective Analysis. J. Clin. Med..

[13] Costanzo A., Bianchi L., Flori M.L., Malara G., Stingeni L., Bartezaghi M., Carraro L., Castellino G., SUPREME Study Group (2018). Secukinumab Shows High Efficacy Irrespective of *HLA-Cw6* Status in Patients with Moderate-to-Severe Plaque-Type Psoriasis: SUPREME Study. Br. J. Dermatol..

[14] Guðjónsson J.E., Kárason A., Antonsdóttir A.A., Rúnarsdóttir E.H., Gulcher J.R., Stefánsson K., Valdimarsson H. (2002). HLA-Cw6-Positive and HLA-Cw6-Negative Patients with Psoriasis Vulgaris have Distinct Clinical Features. J. Investig. Dermatol..

[15] Zhang X., Lei L., Jiang L., Fu C., Huang J., Hu Y., Zhu L., Zhang F., Chen J., Zeng Q. (2023). Characteristics and Pathogenesis of Koebner Phenomenon. Exp. Dermatol..

[16] Owczarek W. (2022). The Role of HLA-Cw6 in Psoriasis and Psoriatic Arthritis. Rheumatology.

[17] Liu P., Kuang Y., Ye L., Peng C., Chen W., Shen M., Zhang M., Zhu W., Lv C., Chen X. (2021). Predicting the Risk of Psoriatic Arthritis in Plaque Psoriasis Patients: Development and Assessment of a New Predictive Nomogram. Front. Immunol..

[18] Wilson F.C., Icen M., Crowson C.S., McEvoy M.T., Gabriel S.E., Kremers H.M. (2009). Incidence and Clinical Predictors of Psoriatic Arthritis in Patients with Psoriasis: A Population-Based Study. Arthritis Care Res..

[19] Mallon E., Bunce M., Savoie H., Rowe A., Newson R., Gotch F., Bunker C.B. (2000). HLA-C and Guttate Psoriasis. Br. J. Dermatol..

[20] Bachelez H. (2020). Pustular Psoriasis: The Dawn of a New Era. Acta Derm.-Venereol..

[21] Onoufriadis A., Simpson M.A., Pink A.E., Di Meglio P., Smith C.H., Pullabhatla V., Knight J., Spain S.L., Nestle F.O., Burden A.D. (2011). Mutations in IL36RN/IL1F5 Are Associated with the Severe Episodic Inflammatory Skin Disease Known as Generalized Pustular Psoriasis. Am. J. Hum. Genet..

[22] Twelves S., Mostafa A., Dand N., Burri E., Farkas K., Wilson R., Cooper H.L., Irvine A.D., Oon H.H., Kingo K. (2019). Clinical and Genetic Differences between Pustular Psoriasis Subtypes. J. Allergy Clin. Immunol..

[23] Schön M.P., Erpenbeck L. (2018). The Interleukin-23/Interleukin-17 Axis Links Adaptive and Innate Immunity in Psoriasis. Front. Immunol..

[24] Mössner R., Frambach Y., Wilsmann-Theis D., Löhr S., Jacobi A., Weyergraf A., Müller M., Philipp S., Renner R., Traupe H. (2015). Palmoplantar Pustular Psoriasis Is Associated with Missense Variants in CARD14, but Not with Loss-of-Function Mutations in IL36RN in European Patients. J. Investig. Dermatol..

[25] Raharja A., Mahil S.K., Barker J.N. (2021). Psoriasis: A brief overview. Clin. Med..

[26] Dopytalska K., Ciechanowicz P., Wiszniewski K., Szymańska E., Walecka I. (2021). The Role of Epigenetic Factors in Psoriasis. Int. J. Mol. Sci..

[27] Natoli V., Charras A., Hofmann S.R., Northey S., Russ S., Schulze F., McCann L., Abraham S., Hedrich C.M. (2023). DNA Methylation Patterns in CD4+ T-Cells Separate Psoriasis Patients from Healthy Controls, and Skin Psoriasis from Psoriatic Arthritis. Front. Immunol..

[28] Zeng C., Tsoi L.C., Gudjonsson J.E. (2021). Dysregulated Epigenetic Modifications in Psoriasis. Exp. Dermatol..

[29] Ovejero-Benito M.C., Reolid A., Sánchez-Jiménez P., Saiz-Rodríguez M., Muñoz-Aceituno E., Llamas-Velasco M., Martín-Vilchez S., Cabaleiro T., Román M., Ochoa D. (2018). Histone Modifications Associated with Biological Drug Response in Moderate-to-Severe Psoriasis. Exp. Dermatol..

[30] Domingo S., Solé C., Moliné T., Ferrer B., Cortés-Hernández J. (2020). MicroRNAs in Several Cutaneous Autoimmune Diseases: Psoriasis, Cutaneous Lupus Erythematosus and Atopic Dermatitis. Cells.

[31] Arakawa A., Reeves E., Vollmer S., Arakawa Y., He M., Galinski A., Stöhr J., Dornmair K., James E., Prinz J.C. (2021). ERAP1 Controls the Autoimmune Response against Melanocytes in Psoriasis by Generating the Melanocyte Autoantigen and Regulating Its Amount for HLA-C*06:02 Presentation. J. Immunol..

[32] Swindell W.R., Beamer M.A., Sarkar M.K., Loftus S., Fullmer J., Xing X., Ward N.L., Tsoi L.C., Kahlenberg M.J., Liang Y. (2018). RNA-Seq Analysis of IL-1B and IL-36 Responses in Epidermal Keratinocytes Identifies a Shared MyD88-Dependent Gene Signature. Front. Immunol..

[33] Raychaudhuri S.P., Jiang W.-Y., Raychaudhuri S.K. (2008). Revisiting the Koebner Phenomenon: Role of NGF and Its Receptor System in the Pathogenesis of Psoriasis. Am. J. Pathol..

[34] Ryan G.E., Harris J.E., Richmond J.M. (2021). Resident Memory T Cells in Autoimmune Skin Diseases. Front. Immunol..

[35] Zhang L.-J. (2019). Type1 Interferons Potential Initiating Factors Linking Skin Wounds with Psoriasis Pathogenesis. Front. Immunol..

[36] Arck P.C., Slominski A., Theoharides T.C., Peters E.M.J., Paus R. (2006). Neuroimmunology of Stress: Skin Takes Center Stage. J. Investig. Dermatol..

[37] Martins A.M., Ascenso A., Ribeiro H.M., Marto J. (2020). The Brain–Skin Connection and the Pathogenesis of Psoriasis: A Review with a Focus on the Serotonergic System. Cells.

[38] Zhou S., Yao Z. (2022). Roles of Infection in Psoriasis. Int. J. Mol. Sci..

[39] Nielsen M.B., Ødum N., Gerwien J., Svejgaard A., Bendtzen K., Bregentholt S., Rōpke C., Geisler C., Dohlsten M., Kaltoft K. (1998). Staphylococcal Enterotoxin-A Directly Stimulates Signal Transduction and Interferon-γ production in Psoriatic T-Cell Lines. Tissue Antigens.

[40] Morar N.A., Willis-Owen S.A., Maurer T., Bunker C.B. (2010). HIV-Associated Psoriasis: Pathogenesis, Clinical Features, and Management. Lancet Infect. Dis..

[41] Fife D.J., Waller J.M., Jeffes E.W., Koo J.Y.M. (2007). Unraveling the Paradoxes of HIV-Associated Psoriasis: A Review of T-Cell Subsets and Cytokine Profiles. Dermatol. Online J..

[42] Alpalhão M., Borges-Costa J., Filipe P. (2019). Psoriasis in HIV Infection: An Update. Int. J. STD AIDS.

[43] Teng Y., Xie W., Tao X., Liu N., Yu Y., Huang Y., Xu D., Fan Y. (2021). Infection-Provoked Psoriasis: Induced or Aggravated (Review). Exp. Ther. Med..

[44] Tsai Y.-C., Tsai T.-F. (2019). A Review of Antibiotics and Psoriasis: Induction, Exacerbation, and Amelioration. Expert Rev. Clin. Pharmacol..

[45] Polak K., Bergler-Czop B., Szczepanek M., Wojciechowska K., Frątczak A., Kiss N. (2021). Psoriasis and Gut Microbiome—Current State of Art. Int. J. Mol. Sci..

[46] Ramírez-Boscá A., Navarro-López V., Martínez-Andrés A., Such J., Francés R., de la Parte J.H., Asín-Llorca M. (2015). Identification of Bacterial DNA in the Peripheral Blood of Patients with Active Psoriasis. JAMA Dermatol..

[47] Armstrong A.W., Read C. (2020). Pathophysiology, Clinical Presentation, and Treatment of Psoriasis: A Review. JAMA.

[48] van de Kerkhof P.C.M., Franssen M.E.J. (2001). Psoriasis of the Scalp. Diagnosis and Management. Am. J. Clin. Dermatol..

[49] Papp K., Berth-Jones J., Kragballe K., Wozel G., de la Brassinne M. (2007). Scalp Psoriasis: A Review of Current Topical Treatment Options. J. Eur. Acad. Dermatol. Venereol..

[50] Reich K. (2009). Approach to Managing Patients with Nail Psoriasis. J. Eur. Acad. Dermatol. Venereol..

[51] Jiaravuthisan M.M., Sasseville D., Vender R.B., Murphy F., Muhn C.Y. (2007). Psoriasis of the Nail: Anatomy, Pathology, Clinical Presentation, and a Review of the Literature on Therapy. J. Am. Acad. Dermatol..

[52] Raychaudhuri S.K., Maverakis E., Raychaudhuri S.P. (2014). Diagnosis and Classification of Psoriasis. Autoimmun. Rev..

[53] Murphy M., Kerr P., Grant-Kels J.M. (2007). The Histopathologic Spectrum of Psoriasis. Clin. Dermatol..

[54] Abdou A.G., Hammam M., Saad E., Hassan R.A.A. (2022). The Significance of Endocan Immunohistochemical Expression in Chronic Plaque Psoriasis. J. Cosmet. Dermatol..

[55] Kastelan M., Massari L.P., Gruber F., Zamolo G., Zauhar G., Coklo M., Rukavina D. (2004). Perforin Expression Is Upregulated in the Epidermis of Psoriatic Lesions. Br. J. Dermatol..

[56] Simonetti O., Lucarini G., Goteri G., Zizzi A., Biagini G., Muzio L.L., Offidani A. (2006). VEGF is Likely a Key Factor in the Link between Inflammation and Angiogenesis in Psoriasis: Results of an Immunohistochemical Study. Int. J. Immunopathol. Pharmacol..

[57] Hemida A.S., Mareae A.H., Elbasiony A.S.A., Shehata W.A. (2020). Plexin-B2 in Psoriasis; a Clinical and Immunohistochemical Study. J. Immunoass. Immunochem..

[58] Zhang C., Xiao C., Dang E., Cao J., Zhu Z., Fu M., Yao X., Liu Y., Jin B., Wang G. (2018). CD100–Plexin-B2 Promotes the Inflammation in Psoriasis by Activating NF-κB and the Inflammasome in Keratinocytes. J. Investig. Dermatol..

[59] Di Fusco D., Laudisi F., Dinallo V., Monteleone I., Di Grazia A., Marafini I., Troncone E., Colantoni A., Ortenzi A., Stolfi C. (2017). Smad7 Positively Regulates Keratinocyte Proliferation in Psoriasis. Br. J. Dermatol..

[60] Wang X., Sun X., Qu X., Li C., Yang P., Jia J., Liu J., Zheng Y. (2019). Overexpressed Fibulin-3 Contributes to the Pathogenesis of Psoriasis by Promoting Angiogenesis. Clin. Exp. Dermatol..

[61] Hemida A.S., Hammam M.A., Salman A.T.A., Shehata W.A. (2020). Smad7 in Psoriasis Vulgaris Patients: A Clinical and Immunohistochemical Study. J. Cosmet. Dermatol..

[62] Uzuncakmak T.K., Karadag A.S., Ozkanli S., Akbulak O., Ozlu E., Akdeniz N., Oguztuzun S. (2020). Alteration of Tissue Expression of Human Beta Defensin-1 and Human Beta Defensin-2 in Psoriasis Vulgaris Following Phototherapy. Biotech. Histochem..

[63] Younes S.F., Bakry O.A. (2016). Immunohistochemical Evaluation of Role of Serotonin in Pathogenesis of Psoriasis. J. Clin. Diagn. Res..

[64] Lacarrubba F., Pellacani G., Gurgone S., Verzì A.E., Micali G. (2015). Advances in Non-Invasive Techniques as Aids to the Diagnosis and Monitoring of Therapeutic Response in Plaque Psoriasis: A Review. Int. J. Dermatol..

[65] Lacarrubba F., Musumeci M.L., Ferraro S., Stinco G., Verzì A.E., Micali G. (2016). A Three-Cohort Comparison with Videodermatoscopic Evidence of the Distinct Homogeneous Bushy Capillary Microvascular Pattern in Psoriasis vs Atopic Dermatitis and Contact Dermatitis. J. Eur. Acad. Dermatol. Venereol..

[66] Lallas A., Kyrgidis A., Tzellos T.G., Apalla Z., Karakyriou E., Karatolias A., Lefaki I., Sotiriou E., Ioannides D., Argenziano G. (2012). Accuracy of Dermoscopic Criteria for the Diagnosis of Psoriasis, Dermatitis, Lichen Planus and Pityriasis Rosea. Br. J. Dermatol..

[67] Golińska J., Sar-Pomian M., Rudnicka L. (2021). Dermoscopy of Plaque Psoriasis Differs with Plaque Location, Its Duration, and Patient’s Sex. Ski. Res. Technol..

[68] Errichetti E., Stinco G. (2018). Clinical and Dermoscopic Response Predictors in Psoriatic Patients Undergoing Narrowband Ultraviolet B Phototherapy: Results from a Prospective Study. Int. J. Dermatol..

[69] Lallas A., Argenziano G., Zalaudek I., Apalla Z., Ardigo M., Chellini P., Cordeiro N., Guimaraes M., Kyrgidis A., Lazaridou E. (2016). Dermoscopic Hemorrhagic Dots: An Early Predictor of Response of Psoriasis to Biologic Agents. Dermatol. Pract. Concept..

[70] Vergilio M.M., Monteiro E Silva S.A., Jales R.M., Leonardi G.R. (2021). High-Frequency Ultrasound as a Scientific Tool for Skin Imaging Analysis. Exp. Dermatol..

[71] Șomlea M.C., Boca A.N., Pop A.D., Ilieș R.F., Vesa S.C., Buzoianu A.D., Tătaru A. (2019). High-Frequency Ultrasonography of Psoriatic Skin: A Non-Invasive Technique in the Evaluation of the Entire Skin of Patients with Psoriasis: A Pilot Study. Exp. Ther. Med..

[72] Grajdeanu I.-A., Statescu L., Vata D., Popescu I.A., Porumb-Andrese E., Patrascu A.I., Taranu T., Crisan M., Solovastru L.G. (2019). Imaging Techniques in the Diagnosis and Monitoring of Psoriasis. Exp. Ther. Med..

[73] Gutierrez M., Wortsman X., Filippucci E., De Angelis R., Filosa G., Grassi W. (2009). High-Frequency Sonography in the Evaluation of Psoriasis: Nail and Skin Involvement. J. Ultrasound Med..

[74] Lacarrubba F., Nardone B., Musumeci M.L., Micali G. (2009). Ultrasound Evaluation of Clobetasol Propionate 0.05% Foam Application in Psoriatic and Healthy Skin: A Pilot Study. Dermatol. Ther..

[75] Polańska A., Gaura T., Bowszyc-Dmochowska M., Osmola-Mańkowska A., Olek-Hrab K., Adamski Z., Żaba R., Dańczak-Pazdrowska A. (2019). Calcipotriol/Betamethasone Ointment Compared to Narrow-Band UVB in Plaque Psoriasis: First Clinical and Ultrasonographic Study. Int. J. Dermatol..

[76] Dini V., Janowska A., Faita F., Panduri S., Benincasa B.B., Izzetti R., Romanelli M., Oranges T. (2021). Ultra-High-Frequency Ultrasound Monitoring of Plaque Psoriasis during Ixekizumab Treatment. Ski. Res. Technol..

[77] Gutierrez M., De Angelis R., Bernardini M.L., Filippucci E., Goteri G., Brandozzi G., Lemme G., Campanati A., Grassi W., Offidani A. (2011). Clinical, Power Doppler Sonography and Histological Assessment of the Psoriatic Plaque: Short-Term Monitoring in Patients Treated with Etanercept. Br. J. Dermatol..

[78] Calzavara-Pinton P., Longo C., Venturini M., Sala R., Pellacani G. (2008). Reflectance Confocal Microscopy for In Vivo Skin Imaging^†^. Photochem. Photobiol..

[79] Longo C., Zalaudek I., Argenziano G., Pellacani G. (2012). New Directions in Dermatopathology: In Vivo Confocal Microscopy in Clinical Practice. Dermatol. Clin..

[80] Ardigo M., Cota C., Berardesca E., González S. (2009). Concordance between In Vivo Reflectance Confocal Microscopy and Histology in the Evaluation of Plaque Psoriasis. J. Eur. Acad. Dermatol. Venereol..

[81] Zhong L.-S., Wei Z.-P., Liu Y.-Q. (2012). Sensitivity and Specificity of Munro Microabscess Detected by Reflectance Confocal Microscopy in the Diagnosis of Psoriasis Vulgaris. J. Dermatol..

[82] Wolberink E.a.W., van Erp P.E.J., de Boer-van Huizen R.T., van de Kerkhof P.C.M., Gerritsen M.J.P. (2012). Reflectance Confocal Microscopy: An Effective Tool for Monitoring Ultraviolet B Phototherapy in Psoriasis. Br. J. Dermatol..

[83] Ardigò M., Agozzino M., Longo C., Lallas A., Di Lernia V., Fabiano A., Conti A., Sperduti I., Argenziano G., Berardesca E. (2015). Reflectance Confocal Microscopy for Plaque Psoriasis Therapeutic Follow-up during an Anti-TNF-α Monoclonal Antibody: An Observational Multicenter Study. J. Eur. Acad. Dermatol. Venereol..

[84] Gladkova N.D., Petrova G.A., Nikulin N.K., Radenska-Lopovok S.G., Snopova L.B., Chumakov Y.P., Nasonova V.A., Gelikonov V.M., Gelikonov G.V., Kuranov R.V. (2000). In Vivo Optical Coherence Tomography Imaging of Human Skin: Norm and Pathology. Ski. Res. Technol..

[85] Welzel J., Bruhns M., Wolff H.H. (2003). Optical Coherence Tomography in Contact Dermatitis and Psoriasis. Arch. Dermatol. Res..

[86] Guida S., Longhitano S., Ardigò M., Pampena R., Ciardo S., Bigi L., Mandel V.D., Vaschieri C., Manfredini M., Pezzini C. (2022). Dermoscopy, Confocal Microscopy and Optical Coherence Tomography Features of Main Inflammatory and Autoimmune Skin Diseases: A Systematic Review. Australas. J. Dermatol..

[87] Verzì A., Broggi G., Micali G., Sorci F., Caltabiano R., Lacarrubba F. (2022). Line-Field Confocal Optical Coherence Tomography of Psoriasis, Eczema and Lichen Planus: A Case Series with Histopathological Correlation. J. Eur. Acad. Dermatol. Venereol..

[88] Ha-Wissel L., Yasak H., Huber R., Zillikens D., Ludwig R.J., Thaçi D., Hundt J.E. (2022). Case Report: Optical Coherence Tomography for Monitoring Biologic Therapy in Psoriasis and Atopic Dermatitis. Front. Med..

[89] Morsy H., Kamp S., Thrane L., Behrendt N., Saunder B., Zayan H., Elmagid E.A., Jemec G.B.E. (2010). Optical Coherence Tomography Imaging of Psoriasis Vulgaris: Correlation with Histology and Disease Severity. Arch. Dermatol. Res..

[90] Abignano G., Laws P., Del Galdo F., Marzo-Ortega H., McGonagle D. (2019). Three-Dimensional Nail Imaging by Optical Coherence Tomography: A Novel Biomarker of Response to Therapy for Nail Disease in Psoriasis and Psoriatic Arthritis. Clin. Exp. Dermatol..

[91] Aydin S.Z., Castillo-Gallego C., Ash Z.R., Abignano G., Marzo-Ortega H., Wittmann M., Del Galdo F., McGonagle D. (2013). Potential Use of Optical Coherence Tomography and High-Frequency Ultrasound for the Assessment of Nail Disease in Psoriasis and Psoriatic Arthritis. Dermatology.

[92] Berekméri A., Tiganescu A., Alase A.A., Vital E., Stacey M., Wittmann M. (2019). Non-invasive Approaches for the Diagnosis of Autoimmune/Autoinflammatory Skin Diseases—A Focus on Psoriasis and Lupus erythematosus. Front. Immunol..

[93] Portugal-Cohen M., Kohen R. (2013). Non-Invasive Evaluation of Skin Cytokines Secretion: An Innovative Complementary Method for Monitoring Skin Disorders. Methods.

[94] Matsunaga Y., Hashimoto Y., Ishiko A. (2021). Stratum Corneum Levels of Calprotectin Proteins S100A8/A9 Correlate with Disease Activity in Psoriasis Patients. J. Dermatol..

[95] Orro K., Salk K., Abram K., Arshavskaja J., Meikas A., Karelson M., Neuman T., Kingo K., Spee P. (2023). Assessment of Soluble Skin Surface Protein Levels for Monitoring Psoriasis Vulgaris in Adult Psoriasis Patients Using Non-Invasive Transdermal Analysis Patch: A Pilot Study. Front. Med..

[96] Valenzuela F., Fernández J., Jiménez C., Cavagnola D., Mancilla J.F., Astorga J., Hernández M., Fernández A. (2021). Identification of IL-18 and Soluble Cell Adhesion Molecules in the Gingival Crevicular Fluid as Novel Biomarkers of Psoriasis. Life.

[97] Ganzetti G., Campanati A., Santarelli A., Sartini D., Molinelli E., Brisigotti V., Di Ruscio G., Bobyr I., Emanuelli M., Offidani A. (2016). Salivary Interleukin-1β: Oral Inflammatory Biomarker in Patients with Psoriasis. J. Int. Med. Res..

[98] Gooderham M.J., Papp K.A., Lynde C.W. (2018). Shifting the Focus—The Primary Role of IL-23 in Psoriasis and Other Inflammatory Disorders. J. Eur. Acad. Dermatol. Venereol..

[99] Hawkes J.E., Yan B.Y., Chan T.C., Krueger J.G. (2018). Discovery of the IL-23/IL-17 Signaling Pathway and the Treatment of Psoriasis. J. Immunol..

[100] Nair R.P., Duffin K.C., Helms C., Ding J., Stuart P.E., Goldgar D., Gudjonsson J.E., Li Y., Tejasvi T., Feng B.J. (2009). Genome-Wide Scan Reveals Association of Psoriasis with IL-23 and NF-κB Pathways. Nat. Genet..

[101] Nair R.P., Ruether A., Stuart P.E., Jenisch S., Tejasvi T., Hiremagalore R., Schreiber S., Kabelitz D., Lim H.W., Voor-hees J.J. (2008). Polymorphisms of the IL12B and IL23R Genes Are Associated with Psoriasis. J. Investig. Dermatol..

[102] Boutet M.-A., Nerviani A., Gallo Afflitto G., Pitzalis C. (2018). Role of the IL-23/IL-17 Axis in Psoriasis and Psoriatic Arthritis: The Clinical Importance of Its Divergence in Skin and Joints. Int. J. Mol. Sci..

[103] Vignali D.A.A., Kuchroo V.K. (2012). IL-12 Family Cytokines: Immunological Playmakers. Nat. Immunol..

[104] TTang C., Chen S., Qian H., Huang W. (2012). Interleukin-23: As a Drug Target for Autoimmune Inflammatory Diseases. Immunology.

[105] Bugaut H., Aractingi S. (2021). Major Role of the IL17/23 Axis in Psoriasis Supports the Development of New Targeted Therapies. Front. Immunol..

[106] Liu T., Li S., Ying S., Tang S., Ding Y., Li Y., Qiao J., Fang H. (2020). The IL-23/IL-17 Pathway in Inflammatory Skin Diseases: From Bench to Bedside. Front. Immunol..

[107] Girolomoni G., Strohal R., Puig L., Bachelez H., Barker J., Boehncke W.H., Prinz J.C. (2017). The Role of IL-23 and the IL-23/TH 17 Immune Axis in the Pathogenesis and Treatment of Psoriasis. J. Eur. Acad. Dermatol. Venereol..

[108] Pastor-Fernández G., Mariblanca I.R., Navarro M.N. (2020). Decoding IL-23 Signaling Cascade for New Therapeutic Opportunities. Cells.

[109] Langrish C.L., McKenzie B.S., Wilson N.J., de Waal Malefyt R., Kastelein R.A., Cua D.J. (2004). IL-12 and IL-23: Master Regulators of Innate and Adaptive Immunity. Immunol. Rev..

[110] Mosca M., Hong J., Hadeler E., Hakimi M., Liao W., Bhutani T. (2021). The Role of IL-17 Cytokines in Psoriasis. ImmunoTargets Ther..

[111] Brembilla N.C., Senra L., Boehncke W.-H. (2018). The IL-17 Family of Cytokines in Psoriasis: IL-17A and Beyond. Front. Immunol..

[112] Ghoreschi K., Balato A., Enerbäck C., Sabat R. (2021). Therapeutics Targeting the IL-23 and IL-17 Pathway in Psoriasis. Lancet.

[113] Grän F., Kerstan A., Serfling E., Goebeler M., Muhammad K. (2020). Current Developments in the Immunology of Psoriasis. Yale J. Biol. Med..

[114] Hawkes J.E., Chan T.C., Krueger J.G. (2017). Psoriasis Pathogenesis and the Development of Novel Targeted Immune Therapie. J. Allergy Clin. Immunol..

[115] Furue M., Furue K., Tsuji G., Nakahara T. (2020). Interleukin-17A and Keratinocytes in Psoriasis. Int. J. Mol. Sci..

[116] Albanesi C., Madonna S., Gisondi P., Girolomoni G. (2018). The Interplay Between Keratinocytes and Immune Cells in the Pathogenesis of Psoriasis. Front. Immunol..

[117] Méhul B., Laffet G., Séraïdaris A., Russo L., Fogel P., Carlavan I., Pernin C., Andres P., Queille-Roussel C., Voegel J.J. (2017). Noninvasive Proteome Analysis of Psoriatic Stratum Corneum Reflects Pathophysiological Pathways and Is Useful for Drug Profiling. Br. J. Dermatol..

[118] Harper E.G., Guo C., Rizzo H., Lillis J.V., Kurtz S.E., Skorcheva I., Purdy D., Fitch E., Iordanov M., Blauvelt A. (2009). Th17 Cytokines Stimulate CCL20 Expression in Keratinocytes in Vitro and in Vivo: Implications for Psoriasis Pathogenesis. J. Investig. Dermatol..

[119] Ni X., Lai Y. (2020). Keratinocyte: A Trigger or an Executor of Psoriasis?. J. Leukoc. Biol..

[120] Jang D.-I., Lee A.-H., Shin H.-Y., Song H.-R., Park J.-H., Kang T.-B., Lee S.-R., Yang S.-H. (2021). The Role of Tumor Necrosis Factor Alpha (TNF-α) in Autoimmune Disease and Current TNF-α Inhibitors in Therapeutics. Int. J. Mol. Sci..

[121] Tan J.K., Aphale A., Malaviya R., Sun Y., Gottlieb A.B. (2007). Mechanisms of Action of Etanercept in Psoriasis. J. Investig. Dermatol. Symp. Proc..

[122] Yost J., Gudjonsson J.E. (2009). The Role of TNF Inhibitors in Psoriasis Therapy: New Implications for Associated Comorbidities. F1000 Med. Rep..

[123] Baliwag J., Barnes D.H., Johnston A. (2015). Cytokines in Psoriasis. Cytokine.

[124] Frucht D.M., Fukao T., Bogdan C., Schindler H., O’Shea J.J., Koyasu S. (2001). IFN-Gamma Production by Antigen-Presenting Cells: Mechanisms Emerge. Trends Immunol..

[125] Belpaire A., van Geel N., Speeckaert R. (2022). From IL-17 to IFN-γ in Inflammatory Skin Disorders: Is Transdifferentiation a Potential Treatment Target?. Front. Immunol..

[126] Tsiogkas S.G., Mavropoulos A., Dardiotis E., Zafiriou E., Bogdanos D.P. (2022). A Sharp Decrease of Th17, CXCR3+-Th17, and Th17.1 in Peripheral Blood Is Associated with an Early Anti-IL-17-Mediated Clinical Remission in Psoriasis. Clin. Exp. Immunol..

[127] Chiricozzi A., Romanelli P., Volpe E., Borsellino G., Romanelli M. (2018). Scanning the Immunopathogenesis of Psoriasis. Int. J. Mol. Sci..

[128] Zhou L., Todorovic V. (2021). Interleukin-36: Structure, Signaling and Function. Adv. Exp. Med. Biol..

[129] Macleod T., Ainscough J.S., Hesse C., Konzok S., Braun A., Buhl A.-L., Wenzel J., Bowyer P., Terao Y., Herrick S. (2020). The Proinflammatory Cytokine IL-36γ Is a Global Discriminator of Harmless Microbes and Invasive Pathogens within Epithelial Tissues. Cell Rep..

[130] Sachen K.L., Arnold Greving C.N., Towne J.E. (2022). Role of IL-36 Cytokines in Psoriasis and Other Inflammatory Skin Conditions. Cytokine.

[131] D’Erme A.M., Wilsmann-Theis D., Wagenpfeil J., Hölzel M., Ferring-Schmitt S., Sternberg S., Wittmann M., Peters B., Bosio A., Bieber T. (2015). IL-36γ (IL-1F9) Is a Biomarker for Psoriasis Skin Lesions. J. Investig. Dermatol..

[132] Tang X., Chen L. (2022). The Risk of Organ-Based Comorbidities in Psoriasis: A Systematic Review and Meta-Analysis. An. Bras. Dermatol..

[133] Bu J., Ding R., Zhou L., Chen X., Shen E. (2022). Epidemiology of Psoriasis and Comorbid Diseases: A Narrative Review. Front. Immunol..

[134] Piepoli M.F., Hoes A.W., Agewall S., Albus C., Brotons C., Catapano A.L., Cooney M.-T., Corrà U., Cosyns B., Deaton C. (2016). 2016 European Guidelines on cardiovascular disease prevention in clinical practice. Kardiol. Pol..

[135] Dey A.K., Joshi A.A., Chaturvedi A., Lerman J.B., Aberra T.M., Rodante J.A., Teague H.L., Harrington C.L., Rivers J.P., Chung J.H. (2017). Association Between Skin and Aortic Vascular Inflammation in Patients With Psoriasis: A Case-Cohort Study Using Positron Emission Tomography/Computed Tomography. JAMA Cardiol..

[136] Egeberg A., Skov L., Joshi A.A., Mallbris L., Gislason G.H., Wu J.J., Rodante J., Lerman J.B., Ahlman M.A., Gelfand J.M. (2017). The Relationship between Duration of Psoriasis, Vascular Inflammation, and Cardiovascular Events. J. Am. Acad. Dermatol..

[137] Hjuler K.F., Bøttcher M., Vestergaard C., Bøtker H.E., Iversen L., Kragballe K. (2016). Association Between Changes in Coronary Artery Disease Progression and Treatment With Biologic Agents for Severe Psoriasis. JAMA Dermatol..

[138] Wu J.J., Guérin A., Sundaram M., Dea K., Cloutier M., Mulani P. (2017). Cardiovascular Event Risk Assessment in Psoriasis Patients Treated with Tumor Necrosis Factor-α Inhibitors versus Methotrexate. J. Am. Acad. Dermatol..

[139] Orlando G., Molon B., Viola A., Alaibac M., Angioni R., Piaserico S. (2022). Psoriasis and Cardiovascular Diseases: An Immune-Mediated Cross Talk?. Front. Immunol..

[140] Elnabawi Y.A., Dey A.K., Goyal A., Groenendyk J.W., Chung J.H., Belur A.D., Rodante J., Harrington C.L., Teague H.L., Baumer Y. (2019). Coronary Artery Plaque Characteristics and Treatment with Biologic Therapy in Severe Psoriasis: Results from a Prospective Observational Study. Cardiovasc. Res..

[141] Sajja A.P., Joshi A.A., Teague H.L., Dey A.K., Mehta N.N. (2018). Potential Immunological Links Between Psoriasis and Cardiovascular Disease. Front. Immunol..

[142] Liu C., Chen H., Liu Y., Huang H., Yu W., Du T., Long X., Chen X., Chen Z., Guo S. (2022). Immunity: Psoriasis Comorbid with Atherosclerosis. Front. Immunol..

[143] Huang P.L. (2009). A Comprehensive Definition for Metabolic Syndrome. Dis. Model. Mech..

[144] Gisondi P., Fostini A.C., Fossà I., Girolomoni G., Targher G. (2018). Psoriasis and the Metabolic Syndrome. Clin. Dermatol..

[145] Wu J.J., Kavanaugh A., Lebwohl M.G., Gniadecki R., Merola J.F. (2022). Psoriasis and Metabolic Syndrome: Implications for the Management and Treatment of Psoriasis. J. Eur. Acad. Dermatol. Venereol..

[146] Hao Y., Zhu Y.-J., Zou S., Zhou P., Hu Y.-W., Zhao Q.-X., Gu L.-N., Zhang H.-Z., Wang Z., Li J. (2021). Metabolic Syndrome and Psoriasis: Mechanisms and Future Directions. Front. Immunol..

[147] Cerman A.A., Bozkurt S., Sav A., Tulunay A., Elbaşi M.O., Ergun T. (2008). Serum Leptin Levels, Skin Leptin and Leptin Receptor Expression in Psoriasis. Br. J. Dermatol..

[148] Puig L. (2017). Cardiometabolic Comorbidities in Psoriasis and Psoriatic Arthritis. Int. J. Mol. Sci..

[149] Abdel-Moneim A., Bakery H.H., Allam G. (2018). The Potential Pathogenic Role of IL-17/Th17 Cells in Both Type 1 and Type 2 Diabetes Mellitus. Biomed. Pharmacother..

[150] Akash M.S.H., Rehman K., Liaqat A. (2018). Tumor Necrosis Factor-Alpha: Role in Development of Insulin Resistance and Pathogenesis of Type 2 Diabetes Mellitus. J. Cell. Biochem..

[151] Fatima N., Faisal S.M., Zubair S., Siddiqui S.S., Moin S., Owais M. (2017). Emerging Role of Interleukins IL-23/IL-17 Axis and Biochemical Markers in the Pathogenesis of Type 2 Diabetes: Association with Age and Gender in Human Subjects. Int. J. Biol. Macromol..

[152] Morrison M.C., Kleemann R. (2015). Role of Macrophage Migration Inhibitory Factor in Obesity, Insulin Resistance, Type 2 Diabetes, and Associated Hepatic Co-Morbidities: A Comprehensive Review of Human and Rodent Studies. Front. Immunol..

[153] Bezdek S., Leng L., Busch H., Mousavi S., Rades D., Dahlke M., Zillikens D., Bucala R., Sadik C.D. (2018). Macrophage Migration Inhibitory Factor (MIF) Drives Murine Psoriasiform Dermatitis. Front. Immunol..

[154] Sun Y., Li Y., Zhang J. (2022). The Causal Relationship between Psoriasis, Psoriatic Arthritis, and Inflammatory Bowel Diseases. Sci. Rep..

[155] Li Y., Guo J., Cao Z., Wu J. (2022). Causal Association Between Inflammatory Bowel Disease and Psoriasis: A Two-Sample Bidirectional Mendelian Randomization Study. Front. Immunol..

[156] Cottone M., Sapienza C., Macaluso F.S., Cannizzaro M. (2019). Psoriasis and Inflammatory Bowel Disease. Dig. Dis..

[157] Fiorino G., Omodei P.D. (2015). Psoriasis and Inflammatory Bowel Disease: Two Sides of the Same Coin?. J. Crohn’s Colitis.

[158] Vlachos C., Gaitanis G., Katsanos K.H., Christodoulou D.K., Tsianos E., Bassukas I.D. (2016). Psoriasis and Inflammatory Bowel Disease: Links and Risks. Psoriasis Targets Ther..

[159] Fauny M., Moulin D., D’Amico F., Netter P., Petitpain N., Arnone D., Jouzeau J.-Y., Loeuille D., Peyrin-Biroulet L. (2020). Paradoxical Gastrointestinal Effects of Interleukin-17 Blockers. Ann. Rheum. Dis..

[160] Eppinga H., Sperna Weiland C.J., Thio H.B., van der Woude C.J., Nijsten T.E.C., Peppelenbosch M.P., Konstantinov S.R. (2016). Similar Depletion of Protective Faecalibacterium Prausnitzii in Psoriasis and Inflammatory Bowel Disease, but Not in Hidradenitis Suppurativa. J. Crohn’s Colitis.

[161] Halioua B., Chelli C., Misery L., Taieb J., Taieb C. (2022). Sleep Disorders and Psoriasis: An Update. Acta Derm.-Venereol..

[162] Ruffilli I., Ragusa F., Benvenga S., Vita R., Antonelli A., Fallahi P., Ferrari S.M. (2017). Psoriasis, Psoriatic Arthritis, and Thyroid Autoimmunity. Front. Endocrinol..

[163] Xue H., Yang Y., Zhang Y., Song S., Zhang L., Ma L., Yang T., Liu H. (2015). Macrophage Migration Inhibitory Factor Interacting with Th17 Cells May Be Involved in the Pathogenesis of Autoimmune Damage in Hashimoto’s Thyroiditis. Mediat. Inflamm..

[164] Kuryłowicz A., Nauman J. (2008). The Role of Nuclear Factor-kappaB in the Development of Autoimmune Diseases: A Link between Genes and Environment. Acta Biochim. Pol..

[165] Mleczko M., Gerkowicz A., Krasowska D. (2022). Chronic Inflammation as the Underlying Mechanism of the Development of Lung Diseases in Psoriasis: A Systematic Review. Int. J. Mol. Sci..

[166] Li X., Kong L., Li F., Chen C., Xu R., Wang H., Peng S., Zhou M., Li B. (2015). Association between Psoriasis and Chronic Obstructive Pulmonary Disease: A Systematic Review and Meta-Analysis. PLoS ONE.

[167] Wi D., Wilson A., Satgé F., Murrell D.F. (2022). Psoriasis and Osteoporosis: A Literature Review. Clin. Exp. Dermatol..

[168] Bellinato F., Gisondi P., Mantovani A., Girolomoni G., Targher G. (2022). Risk of Non-Alcoholic Fatty Liver Disease in Patients with Chronic Plaque Psoriasis: An Updated Systematic Review and Meta-Analysis of Observational Studies. J. Endocrinol. Investig..

[169] Sharquie K.E., Salman H.A., Yaseen A.K. (2017). Psoriasis and Vitiligo Are Close Relatives. Clin. Cosmet. Investig. Dermatol..

[170] Zhu K.-J., Lv Y.-M., Yin X.-Y., Wang Z.-X., Sun L.-D., He S.-M., Cheng H., Hu D.-Y., Zhang Z., Li Y. (2011). Psoriasis Regression Analysis of MHC Loci Identifies Shared Genetic Variants with Vitiligo. PLoS ONE.

[171] Kridin K., Ludwig R.J., Schonmann Y., Damiani G., Cohen A.D. (2020). The Bidirectional Association Between Bullous Pemphigoid and Psoriasis: A Population-Based Cohort Study. Front. Med..

[172] Kridin K., Shani M., Schonmann Y., Fisher S., Shalom G., Comaneshter D., Batat E., Cohen A.D. (2023). Psoriasis and Hidradenitis Suppurativa: A Large-Scale Population-Based Study. J. Am. Acad. Dermatol..

[173] Kaushik S.B., Lebwohl M.G. (2018). Psoriasis: Which Therapy for Which Patient: Psoriasis Comorbidities and Preferred Systemic Agents. J. Am. Acad. Dermatol..

[174] Jiang Y., Chen Y., Yu Q., Shi Y. (2023). Biologic and Small-Molecule Therapies for Moderate-to-Severe Psoriasis: Focus on Psoriasis Comorbidities. BioDrugs.

[175] Strober B., Ryan C., van de Kerkhof P., van der Walt J., Kimball A.B., Barker J., Blauvelt A., International Psoriasis Council Board Members and Councilors (2020). Recategorization of Psoriasis Severity: Delphi Consensus from the International Psoriasis Council. J. Am. Acad. Dermatol..

[176] Mahil S.K., Wilson N., Dand N., Reynolds N.J., Griffiths C.E.M., Emsley R., Marsden A., Evans I., Warren R.B., Stocken D. (2020). Psoriasis Treat to Target: Defining Outcomes in Psoriasis Using Data from a Real-World, Population-Based Cohort Study (the British Association of Dermatologists Biologics and Immunomodulators Register, BADBIR). Br. J. Dermatol..

[177] Brownstone N.D., Hong J., Mosca M., Hadeler E., Liao W., Bhutani T., Koo J. (2021). Biologic Treatments of Psoriasis: An Update for the Clinician. Biol. Targets Ther..

[178] Marotte H., Cimaz R. (2014). Etanercept—TNF Receptor and IgG1 Fc Fusion Protein: Is It Different from Other TNF Blockers?. Expert Opin. Biol. Ther..

[179] Klotz U., Teml A., Schwab M. (2007). Clinical Pharmacokinetics and Use of Infliximab. Clin. Pharmacokinet..

[180] Singh R., Koppu S., Perche P.O., Feldman S.R. (2021). The Cytokine Mediated Molecular Pathophysiology of Psoriasis and Its Clinical Implications. Int. J. Mol. Sci..

[181] Alwawi E.A., Mehlis S.L., Gordon K.B. (2008). Treating Psoriasis with Adalimumab. Ther. Clin. Risk Manag..

[182] Goel N., Stephens S. (2010). Certolizumab Pegol. mAbs.

[183] Veronese F.M., Mero A. (2008). The Impact of PEGylation on Biological Therapies. BioDrugs.

[184] Mariette X., Förger F., Abraham B., Flynn A.D., Moltó A., Flipo R.-M., van Tubergen A., Shaughnessy L., Simpson J., Teil M. (2017). Lack of Placental Transfer of Certolizumab Pegol during Pregnancy: Results from CRIB, a Prospective, Postmarketing, Pharmacokinetic Study. Ann. Rheum. Dis..

[185] Yang K., Oak A.S.W., Elewski B.E. (2021). Use of IL-23 Inhibitors for the Treatment of Plaque Psoriasis and Psoriatic Arthritis: A Comprehensive Review. Am. J. Clin. Dermatol..

[186] Kulig P., Musiol S., Freiberger S.N., Schreiner B., Gyülveszi G., Russo G., Pantelyushin S., Kishihara K., Alessandrini F., Kündig T. (2016). IL-12 Protects from Psoriasiform Skin Inflammation. Nat. Commun..

[187] Blauvelt A., Kimball A.B., Augustin M., Okubo Y., Witte M.M., Capriles C.R., Sontag A., Arora V., Osuntokun O., Strober B. (2022). Efficacy and Safety of Mirikizumab in Psoriasis: Results from a 52-Week, Double-Blind, Placebo-Controlled, Randomized Withdrawal, Phase III Trial (OASIS-1). Br. J. Dermatol..

[188] Zhou L., Wang Y., Wan Q., Wu F., Barbon J., Dunstan R., Gauld S., Konrad M., Leys L., McCarthy R. (2021). A Non-Clinical Comparative Study of IL-23 Antibodies in Psoriasis. mAbs.

[189] Ghazawi F.M., Mahmood F., Kircik L., Poulin Y., Bourcier M., Vender R., Wiseman M.C., Lynde C., Litvinov I.V. (2021). A Review of the Efficacy and Safety for Biologic Agents Targeting IL-23 in Treating Psoriasis With the Focus on Tildrakizumab. Front. Med..

[190] Ten Bergen L.L., Petrovic A., Krogh Aarebrot A., Appel S. (2020). The TNF/IL-23/IL-17 Axis-Head-to-Head Trials Comparing Different Biologics in Psoriasis Treatment. Scand. J. Immunol..

[191] Facheris P., Valenti M., Pavia G., Guanziroli E., Narcisi A., Borroni R.G., Costanzo A. (2020). Brodalumab: A New Way to Inhibit IL-17 in Psoriasis. Dermatol. Ther..

[192] Ruggiero A., Potestio L., Camela E., Fabbrocini G., Megna M. (2022). Bimekizumab for the Treatment of Psoriasis: A Review of the Current Knowledge. Psoriasis Targets Ther..

[193] Torres T., Filipe P. (2015). Small Molecules in the Treatment of Psoriasis. Drug Dev. Res..

[194] Claudia C.-D., María-Elena V.-H., Josué V.-E., María-Carmen B.-C., Alain-Raimundo R.-O., Martha-Estrella G.-P. (2020). Small Molecules under Development for Psoriasis: On the Road to the Individualized Therapies. Arch. Dermatol. Res..

[195] Gao J.C., Wu A.G., Contento M.N., Maher J.M., Cline A. (2022). Apremilast in the Treatment of Plaque Psoriasis: Differential Use in Psoriasis. Clin. Cosmet. Investig. Dermatol..

[196] Caputo V., Strafella C., Cosio T., Lanna C., Campione E., Novelli G., Giardina E., Cascella R. (2021). Pharmacogenomics: An Update on Biologics and Small-Molecule Drugs in the Treatment of Psoriasis. Genes.

[197] Warren R.B., Strober B., Silverberg J.I., Guttman E., Andres P., Felding J., Tutkunkardas D., Kjøller K., Sommer M.O.A., French L.E. (2023). Oral Orismilast: Efficacy and Safety in Moderate-to-Severe Psoriasis and Development of Modified Release Tablets. J. Eur. Acad. Dermatol. Venereol..

[198] Dodson J., Lio P.A. (2022). Biologics and Small Molecule Inhibitors: An Update in Therapies for Allergic and Immunologic Skin Diseases. Curr. Allergy Asthma Rep..

[199] Hoy S.M. (2022). Deucravacitinib: First Approval. Drugs.

[200] Zhang J., Qi F., Dong J., Tan Y., Gao L., Liu F. (2022). Application of Baricitinib in Dermatology. J. Inflamm. Res..

[201] Thakur V., Mahajan R. (2022). Novel Therapeutic Target(s) for Psoriatic Disease. Front. Med..

[202] Papp K.A., Krueger J.G., Feldman S.R., Langley R.G., Thaci D., Torii H., Tyring S., Wolk R., Gardner A., Mebus C. (2016). Tofacitinib, an Oral Janus Kinase Inhibitor, for the Treatment of Chronic Plaque Psoriasis: Long-Term Efficacy and Safety Results from 2 Randomized Phase-III Studies and 1 Open-Label Long-Term Extension Study. J. Am. Acad. Dermatol..

[203] Murphy E.C., Schaffter S.W., Friedman A.J. (2019). Nanotechnology for Psoriasis Therapy. Curr. Dermatol. Rep..

[204] Fereig S.A., El-Zaafarany G.M., Arafa M.G., Abdel-Mottaleb M.M.A. (2020). Tackling the Various Classes of Nano-Therapeutics Employed in Topical Therapy of Psoriasis. Drug Deliv..

[205] Dhiman N., Awasthi R., Sharma B., Kharkwal H., Kulkarni G.T. (2021). Lipid Nanoparticles as Carriers for Bioactive Delivery. Front. Chem..

[206] Viegas J.S.R., Praça F.G., Caron A.L., Suzuki I., Silvestrini A.V.P., Medina W.S.G., Del Ciampo J.O., Kravicz M., Bentley M.V.L.B. (2020). Nanostructured Lipid Carrier Co-Delivering Tacrolimus and TNF-α siRNA as an Innovate Approach to Psoriasis. Drug Deliv. Transl. Res..

[207] Kaur A., Katiyar S.S., Kushwah V., Jain S. (2017). Nanoemulsion Loaded Gel for Topical Co-Delivery of Clobitasol Propionate and Calcipotriol in Psoriasis. Nanomed. Nanotechnol. Biol. Med..

[208] Marepally S., Boakye C.H.A., Patel A.R., Godugu C., Doddapaneni R., Desai P.R., Singh M. (2014). Topical Administration of Dual siRNAs Using Fusogenic Lipid Nanoparticles for Treating Psoriatic-like Plaques. Nanomedicine.

[209] Mascarenhas-Melo F., Carvalho A., Gonçalves M.B.S., Paiva-Santos A.C., Veiga F. (2022). Nanocarriers for the Topical Treatment of Psoriasis—Pathophysiology, Conventional Treatments, Nanotechnology, Regulatory and Toxicology. Eur. J. Pharm. Biopharm..

[210] Bessar H., Venditti I., Benassi L., Vaschieri C., Azzoni P., Pellacani G., Magnoni C., Botti E., Casagrande V., Federici M. (2016). Functionalized Gold Nanoparticles for Topical Delivery of Methotrexate for the Possible Treatment of Psoriasis. Colloids Surf. B Biointerfaces.

[211] Bai F., Zheng W., Dong Y., Wang J., Garstka M.A., Li R., An J., Ma H. (2018). Serum Levels of Adipokines and Cytokines in Psoriasis Patients: A Systematic Review and Meta-Analysis. Oncotarget.

[212] Olejniczak-Staruch I., Narbutt J., Bednarski I., Woźniacka A., Sieniawska J., Kraska-Gacka M., Śmigielski J., Lesiak A. (2020). Interleukin 22 and 6 Serum Concentrations Decrease under Long-Term Biologic Therapy in Psoriasis. Adv. Dermatol. Allergol..

[213] Wang X., Kaiser H., Kvist-Hansen A., McCauley B.D., Skov L., Hansen P.R., Becker C. (2022). IL-17 Pathway Members as Potential Biomarkers of Effective Systemic Treatment and Cardiovascular Disease in Patients with Moderate-to-Severe Psoriasis. Int. J. Mol. Sci..

[214] Dickerson T., Lee B.-I., Montgomery P., Boyd S., Andrade E., Boyce C., Tomich T., Wang Y. (2021). Mind.Px—Personalized Medicine for Psoriasis Biologic Treatment. SKIN J. Cutan. Med..

